# Supplementary figures and images for: Euvichol-plus vaccine campaign coverage during the 2017/2018 cholera outbreak in Lusaka district, Zambia: a cross-sectional descriptive study
Source: BMJ Open. 2023 Oct 5;13(10):e070796. doi: 10.1136/bmjopen-2022-070796 (PMC10565249; doi:10.1136/bmjopen-2022-070796)

**Figure 1: Recruitment algorithm**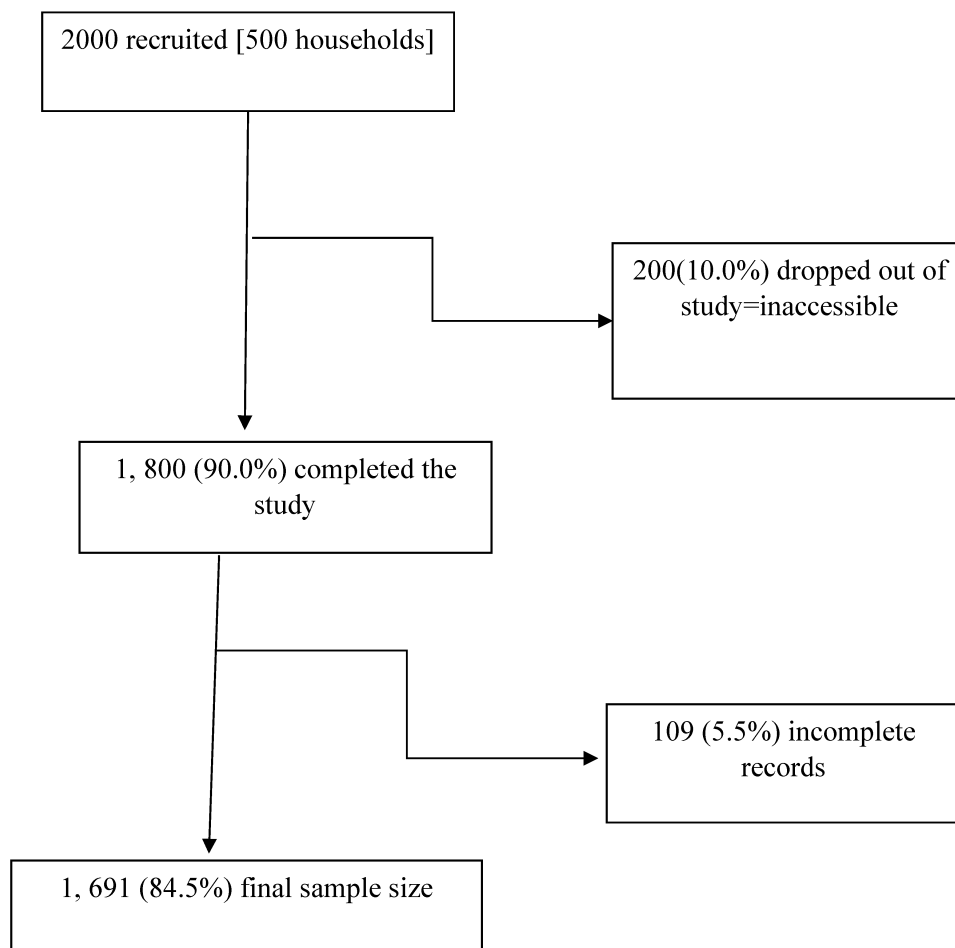

Supplement: Supplementary data [file bmjopen-2022-070796supp001.pdf]
