# Supplementary material for: Euvichol-plus vaccine campaign coverage during the 2017/2018 cholera outbreak in Lusaka district, Zambia: a cross-sectional descriptive study
Source: BMJ Open. 2023 Oct 5;13(10):e070796. doi: 10.1136/bmjopen-2022-070796 (PMC10565249; doi:10.1136/bmjopen-2022-070796)
Supplement: Author's manuscript [file bmjopen-2022-070796.draft_revisions.pdf]

BMJ Open

BMJ Open is committed to open peer review. As part of this commitment we make the peer review history of every article we publish publicly available.

When an article is published we post the peer reviewers' comments and the authors' responses online. We also post the versions of the paper that were used during peer review. These are the versions that the peer review comments apply to.

The versions of the paper that follow are the versions that were submitted during the peer review process. They are not the versions of record or the final published versions. They should not be cited or distributed as the published version of this manuscript.

BMJ Open is an open access journal and the full, final, typeset and author-corrected version of record of the manuscript is available on our site with no access controls, subscription charges or pay-per-view fees (http://bmjopen.bmj.com).

If you have any questions on BMJ Open's open peer review process please email info.bmjopen@bmj.com

BMJ Open

Euvichol-Plus Vaccine Campaign Coverage during the 2017/2018 Cholera outbreak in Lusaka District, Zambia: A Cross Sectional Study

Journal:	*BMJ Open*
Manuscript ID	bmjopen-2022-070796
Article Type:	Original research
Date Submitted by the Author:	05-Dec-2022

Complete List of Authors:	Mukonka, Victor M; Levy Mwanawasa Medical University, School of Public Health; The Copperbelt University, School of Medicine<br>Kapata, Nathan; Zambia National Public Health Institute, 4Epidemic Preparedness and Response, Zambia National Public Health Institute, Lusaka, Zambia<br>Matapo, Belem; World Health Organization, Disease Surveillance<br>Chewe, Orbrie; Zambia National Public Health Institute, Surveillance and Disease Intelligence ; Zambia Ministry of Health, Public Health<br>Ngomah, Albertina; Zambia National Public Health Institute, Communication Information & Research<br>Ngosa, Willaim; Zambia National Public Health Institute, Communication Information and Research<br>Hamoonga, Raymond; Zambia National Public Health Institute, Surveillance and Disease Intelligence<br>Sinyange, Nyambe; Zambia National Public Health Institute, Surveillance and Disease Intelligence<br>Mzyece, Hannah; Zambia National Public Health Institute, Surveillance and Disease Intelligence<br>Mazyanga, Lucy; Zambia National Public Health Institute, Communication Information and Research,<br>Bakyaita, Nathan; World Health Organization<br>Sialubanje, Cephas; Levy Mwanawasa Medical University, School of Public Health

Keywords:	EPIDEMIOLOGY, EPIDEMIOLOGIC STUDIES, INFECTIOUS DISEASES, Tropical medicine < INFECTIOUS DISEASES, PUBLIC HEALTH

SCHOLARONE™
Manuscripts

I, the Submitting Author has the right to grant and does grant on behalf of all authors of the Work (as defined in the below author licence), an exclusive licence and/or a non-exclusive licence for contributions from authors who are: i) UK Crown employees; ii) where BMJ has agreed a CC-BY licence shall apply, and/or iii) in accordance with the terms applicable for US Federal Government officers or employees acting as part of their official duties; on a worldwide, perpetual, irrevocable, royalty-free basis to BMJ Publishing Group Ltd ("BMJ") its licensees and where the relevant Journal is co-owned by BMJ to the co-owners of the Journal, to publish the Work in this journal and any other BMJ products and to exploit all rights, as set out in our licence.

The Submitting Author accepts and understands that any supply made under these terms is made by BMJ to the Submitting Author unless you are acting as an employee on behalf of your employer or a postgraduate student of an affiliated institution which is paying any applicable article publishing charge ("APC") for Open Access articles. Where the Submitting Author wishes to make the Work available on an Open Access basis (and intends to pay the relevant APC), the terms of reuse of such Open Access shall be governed by a Creative Commons licence – details of these licences and which Creative Commons licence will apply to this Work are set out in our licence referred to above.

Other than as permitted in any relevant BMJ Author's Self Archiving Policies, I confirm this Work has not been accepted for publication elsewhere, is not being considered for publication elsewhere and does not duplicate material already published. I confirm all authors consent to publication of this Work and authorise the granting of this licence.

For peer review only

Euvichol-Plus Vaccine Campaign Coverage during the 2017/2018 Cholera outbreak in Lusaka District, Zambia: A Cross Sectional Study

Victor Mukonka[1,2,3]; Cephas Sialubanje[1], Belem Matapo[5]; Orbrie Chewe[3,6]; Albertina Moraes Ngomah[7]; Willaim Ngosa[7], Raymond Hamoonga[3], Nyambe Sinyange[3], Hannah Mzyece[3], Lucy Mazyanga[7], Nathan Bakyaita[5]; * Nathan kapata[4]

[1]School of Public Health, Levy Mwanawasa Medical University, Lusaka, Zambia

[2]School of Medicine, Copperbelt University, Kitwe, Zambia

[3]Surveillance and Disease Intelligence, Zambia National Public Health Institute, Lusaka, Zambia

[4]Epidemic Preparedness and Response, Zambia National Public Health Institute, Lusaka, Zambia

[4]Department of Public Health, Ministry of Health, Lusaka, Zambia

[5]Disease Surveillance, World Health Organisation, Lusaka, Zambia

[6]Department of Public Health, Ministry of Health, Lusaka, Zambia

[7]Communication Information and Research, Zambia National Public Health Institute, Lusaka, Zambia

[8]Laboratory System and Network, Zambia National Public Health Institute, Lusaka, Zambia

Corresponding Author: Cephas Sialubanje

csialubanje@yahoo.com

For peer review only - http://bmjopen.bmj.com/site/about/guidelines.xhtml

Abstract

Objective: To determine the coverage for the oral cholera vaccine (OCV) campaign conducted during the 2017/18 cholera outbreak in Lusaka, Zambia.

Methods: A survey conducted among 1,691 respondents older than 12 months from 369 households following the second round of the 2018 OCV campaign in Kanyama, Chawama, Chipata and Matero sub-districts. A satellite-map based sampling technique was used to randomly select households. A pre-tested electronic questionnaire uploaded on an electronic tablet (ODK Version 1.12.2) was used for data collection. STATA was used for data analysis: descriptive statistics and OCV coverage per dose. Fisher's exact test was used to stratify OCV coverage according to age and sex for each round.

Results

The overall coverage for the first, second and two-doses were 81.3% (95%CI [79.24, 83.36]), 72.1% (95%CI [69.58, 74.62]), and 66% (95% CI [63.22, 68.78], respectively. The drop-out rate was 18.8% (95%CI [14.51, 23.09]). Sex, age and household size were significantly associated with vaccine acceptance. Female participants were 1.5 times more likely to receive the second dose ([AOR]=1.54 (95%CI [1.25, 1.91]). Compared to the children aged 1 to 4 years, those aged 15 to 35 (AOR=0.37, 95% CI (0.52[0.33, 0.82]), 36 to 65 years (AOR=0.65, 95%CI [0.40, 1.08]) and above 65 years (AOR=0.37, 95%CI [0.17, 0.80]) had less odds of receiving the first OCV dose. The odds of receiving the second dose were significantly less among those aged 15 to 35 (AOR=0.63, 95%CI [0.43, 0.92]) and those aged 36 to 65 years (AOR=0.699, 95%CI [0.46,1.062]1).

Conclusion

These findings confirm the 2018 OCV campaign coverage and highlight the need for follow up surveys to validate administrative coverage estimates using population-based methods. Reliance on health facility data alone may mask low coverage and prevent measures to improve programming. Public health interventions should consider sociodemographic factors in order to achieve optimal vaccine coverage.

Word count: 300

For peer review only - http://bmjopen.bmj.com/site/about/guidelines.xhtml

For peer review only

Strengths and weakness

- Conducting the survey shortly after the OCV campaign minimized recall bias
- Verification of the self-reported vaccination status with the vaccination certificate card increased internal validity of the findings

For peer review only - http://bmjopen.bmj.com/site/about/guidelines.xhtml

- Use of a satellite-map based sampling technique to randomly select households reduced selection bias.
- Use of a pre-tested electronic questionnaire uploaded on an electronic tablet using ODK Version 1.12.2. enhanced data quality during field work and analysis
- Although a random satellite-based sampling technique was used to select households, inclusion of all eligible participants found in a household into the study may have introduced selection bias.

For peer review only

1.0 Introduction

1.1. Background

Cholera is a rapidly dehydrating diarrheal illness caused by the toxigenic bacterium *Vibrio cholerae* serogroups O1 and O139 [1]. The 01 serogroup has two biotypes, classical and E1 Tor [2]. During the past

For peer review only - http://bmjopen.bmj.com/site/about/guidelines.xhtml

years, cholera has spread from the Indian subcontinent to involve nearly the whole world and cause pandemics in Africa and South America. Since 1817, the world has experienced seven cholera pandemics, the first six of which were caused by the classical O1 biotype. The current (seventh) pandemic, which began in 1961 and largely confined to Asia and Africa, is mainly caused by the El Tor O1 biotype which has essentially displaced the classical biotype globally since 1993 [2,3]. Over-crowding, poverty, poor sanitation facilities, lack of access to clean and safe water and food contamination increase the risk for cholera outbreaks [4]. Global annual estimates of cholera range from 1.3 to 4. Million cases and 21,000 to 143,000 deaths [5-7]. However, the actual burden of the disease is unknown due to poor reporting, limited surveillance, and lack of laboratory capacity. In Africa, the burden of cholera is mainly in sub-Sahara Africa with approximately 19 countries more affected by epidemics, with 105, 287 cases and 1882 deaths [5-7].

Although access to and provision of safe drinking water, adequate sanitation, disease surveillance, health promotion and case management remain the mainstay of cholera prevention and control [8], use of reasonably priced, safe and effective vaccines can provide an additional intervention for cholera prevention and control [9]. The 2011 World Health Assembly (WHA) [10] resolution 64.15 called for implementation of an integrated and comprehensive approach to cholera control. Consequently, in 2011 the world health organization (WHO) established a global stockpile under the supervision of the International Coordination Group on Vaccine Provision to strengthen the capacity for action against cholera in emergency settings and recommended use of oral cholera vaccines (OCV) in endemic and epidemic settings [11]. Subsequently, four killed, whole-cell OCVs–Dukoral, mORCVAX, Shanchol and Euvichol–are currently available and licensed for use in different countries [11]. Shanchol and Euvichol are killed, whole-cell, bivalent O1 and O139 and licensed for use in children ≥ 1 year and require 2 doses given 2 weeks apart. They are the preferred vaccines for public health use as they do not need a buffer [12].

Numerous studies have demonstrated the efficacy, safety and immunogenicity of the killed whole-cell OCV [13-16]. For example, a randomized placebo controlled safety and immunogenicity study conducted among healthy adults in cholera endemic area in Kolkata, India [17] found that the reformulated oral killed whole cell vaccine was safe, well tolerated and highly immunogenic. Studies have also shown that Euvichol has the same formulation as Shanchol and that it has non-inferior sero-conversion rates to those of Shanchol [18]. The study also reported a fourfold rise in serum *v.cholera* 01 vibriocidal antibody titers among 53% adults and 80% children.

For peer review only - http://bmjopen.bmj.com/site/about/guidelines.xhtml

Following the 2017/18 cholera outbreaks [19] and based on lessons from the previous outbreaks [20], the Zambian Ministry of Health (MoH) through WHO and with support from the global task force on cholera control (GFTCC), procured 2,070,100 vaccine doses of the Euvichol-Plus™ OCV campaign to support the short- to-medium term interventions to control cholera. The first OCV round was launched on the 10th January 2018 in the four affected sub-districts of Lusaka (that is, Chawama, Matero, Kanyama and Chipata). In addition to the OCV campaign, the Zambian MOH implemented the following interventions: provision of safe water, chlorination of unsafe water sources, improved sanitation and solid waste management, training of community workers, cleaning of the environment with community involvement and health promotion campaigns through door to door campaigns, public address system and mass media (radio and television).

1.2. Objectives

The aim of this study was to determine the actual OCV coverage for the 2018 OCV campaign conducted in Lusaka district. Although administrative coverage data was collected and recorded during the campaign, information on the actual vaccination coverage (based on the population vaccinated) is required to validate the administrative coverage that was reported at 109% for the first round. Information is also important for planning of future interventions and providing the basis for monitoring and evaluation of activities for future OCV campaigns in Zambia. It is also necessary to evaluate vaccine uptake in the selected target population, identify vaccine acceptability, barriers to vaccination, and any adverse events reported after OCV vaccination. Currently, there is a lack of evidence on the coverage of OCV campaigns and its acceptability among the Zambian population.

2. Methods

2.1. Study design

This was a survey conducted among 1,691 respondents from 369 households following the second round of the 2018 OCV campaign in the four subdistricts of Lusaka, Zambia.

2.2. Study site and population

The study sites included the four sub-districts of Lusaka (Kanyama, Chawama, Chipata and Matero) where the 2018 OCV campaign was conducted (see our previous work on the 2018 Lusaka cholera outbreak and

For peer review only - http://bmjopen.bmj.com/site/about/guidelines.xhtml

for the details on the study setting [22-24]. The study population included all individuals older than 12 months residing in the study sites

2.3. Inclusion criteria

To be eligible to participate in the survey, respondents needed to:

a) Be residing in study sites for at least two weeks prior to the last date of vaccine administration.
b) Older than 12 months
c) Provide consent to participate in the study

2.4. Sample size determination

To estimate the sample size for the survey, the following assumptions were made: a) 50% of the population received two doses; b) confidence interval of 95% (equivalent to an alpha error of 5%); precision of 5; c) design effect of 3; and d) 15% missing data and non-response (18,19). This gave an estimated sample size of 1, 325 of respondents. To estimate the required number of households, the Zambia Demographic and Health Survey report of 2013-14 [25] which showed a mean number of 4 persons per household was used. This gave a total of 332 households (1325/4=331.25) to be included in the study. A household was defined as a group of people sleeping under the same roof and sharing meals every day for at least two previous weeks.

Sampling techniques

A random satellite-based sampling technique was used to select households for inclusion in the study. First, a random point was generated in the sampling area and retained if it coincided with a household (+/- 10 meters error); the process was repeated until the required sample size was reached. All eligible individuals in the selected household at the time of the survey were selected.

2.5. Data Collection

Twenty four (24) trained and experienced research assistants were recruited from the previously conducted coverage survey in Lusaka district. Research assistants were trained for four days; the training comprised classroom-based theory for 3 three days followed by field work for one day. Topics covered during classroom

For peer review only

For peer review only - http://bmjopen.bmj.com/site/about/guidelines.xhtml

training included the a) purpose of the study, b) study methods including sampling techniques; and d) electronic questionnaire administration. Field work included testing the data collection tools, household location using geographical positioning system (GPS) technique. Data collection instruments were revised based on feedback from the research assistants.

Research assistants worked in teams of four, comprising three members and a team leader. Data collection techniques comprised face to face interview (so that they understand the number and proportion of people that received the vaccine in the household), collection of GPS coordinates followed by collection of data on health promotion and cholera prevention. Face to face interview was conducted in the local language. A second visit was organized later in the day or in a second day if responders were not found at the house. The household was skipped if respondents were not found or refused to participate during the second visit.

2.6. Data Collection Instruments

A pre-tested electronic questionnaire uploaded on the electronic tablet using ODK Version 1.12.2 was used to collect data during face to face interviews. The questionnaire comprised questions on eligibility, socio-demographic data (age, sex and household size), vaccination status (self-reported and card-confirmed) and acceptability data (perception and beliefs about the vaccine, vaccination adverse events). To ensure quality in the data collection process, the team leader cross checked the completed questionnaires on a daily basis. Completed and verified questionnaires were transmitted to the server at Zambia National Public Health Institute.

2.7. Quality assurance

To ensure quality assurance in the data collection process, the following measures were taken: a) the survey was conducted about 2 weeks after completion of the second round of the vaccination campaign to minimise recall bias; b) research assistants were trained on data collection methods; c) an electronic data collection tool uploaded on the ODL kit was pre-tested and revised accordingly; c) field supervisors worked with the research assistants and verified the data entry before the data was transmitted to the sever; d) research assistants had no access to the transmitted data; e) householders who were not present during the field were revisited to avoid selection bias.

2.8. Data Processing and Analysis

For peer review only - http://bmjopen.bmj.com/site/about/guidelines.xhtml

Data was retrieved from the server, cleaned and saved on an excel sheet. Analysis was done using STATA version 12.4. Descriptive statistics were computed to summarise respondent and household characteristics and OCV campaign coverage for each round**.** Vaccination status was determined using both the vaccination certificate (card-confirmed) and self-reports. In addition, Fisher's exact test was used to compute the OCV vaccine coverage by sex and age group for each round (95% Confidence Interval, $p<0.005$). To estimate coverage of the 2018 vaccination campaign, the following indicators were computed: a) vaccine coverage for two doses, b) vaccine coverage for the first round; c) vaccine coverage for the second round; d) vaccine coverage with at least one dose. Vaccine coverage was calculated by dividing the number of individuals reporting being vaccinated by the survey population and expressed as a percentage. In addition, the drop-out rate the two rounds was calculated as shown below:

$$Drop\ out\ rate = \left(\frac{Vaccin\_1 - Vaccin\_1\&\_2}{Vaccin\_1} \times 100\right)$$

Vaccin_1 = the number of people vaccinated during the 1st round.

Vaccin_1& 2 = the number of people vaccinated during the 1st and the 2nd round.

2.9. Patient and Public Involvement

The study design was determined by the research team. Participants and the public were not directly involved in the conceptualisation and design of the study. However, selection of the study sites was done in consultation with stakeholders from the Ministry of Health and Zambia National Public Health Institute. Selection of study participants was done in collaboration with the provincial and district health managers. A dissemination meeting was held in Lusaka and study findings shared with key stakeholders, including the World Health Organisation, Ministry of Health, and community leaders in the health facilities where the study was conducted. A final report was also written and shared with the funding organization.

2.10. Ethical considerations

Before each interview, research assistants explained the purpose of the study to the respondents. To ensure privacy for participants, interviews were conducted in the house away from the public interference. To ensure confidentiality, all data were entered and analyzed anonymously. In addition, it was explained that the dataset would be kept on a password-protected computer by the data manager at ZNPHI. Apart from the

For peer review only - http://bmjopen.bmj.com/site/about/guidelines.xhtml

purpose of the study, research assistants explained that the study had no direct benefits on the participants. Rather, the information gained from the study would help improve planning for future interventions and provide basis for monitoring and evaluation of activities for future OCV campaigns in Zambia. The research assistant also explained that the study had no potential risks to participants and that participation in the study was voluntary. Study participants were also advised that they were free to withdraw from the interview at any time. Next, respondents were provided with an opportunity to ask questions so they could make an informed decision on participation into the study. Participation into the study was only allowed after consent was obtained. Finally, written informed consent (**supplementary material 1**) was obtained from each individual (or assent in the case of a minor, with permission from the guardian of the household visited) participate in the study. Those who could not write were asked to use their thumb or mark with an 'X'.

3. Results

Participants

A total of 2,000 participants were recruited into the study. Of these, 200 (10%) could not be traced due to access challenges in the urban slums and thus dropped out of the study. A total of 1800 (90%) participants completed the study, out of whom 109 (5.5%) had incomplete records and were not included in the final analysis. A total final sample of 1, 691 respondents (84.5%) were included in the final analysis. A summary of the recruitment algorithm of study participants is shown in figure 1 (**supplementary material 2**)

Demographic characteristics.

Most respondents were female (58.8%) and aged below 30 (53.3%). Slightly more than two fifths (43.6%) of the respondents had completed primary education, 36.3% had completed secondary education and 5.3% had attended tertiary education and 14.7% never attended school. With regard to occupation, 16.0% were in formal employment, 40.3% were in informal employment and 40.3% were unemployed (**Table 1**).

Table 1: Demographic characteristics

Variable	n (%)
**Sex**	
Male	697 (41.2)

For peer review only - http://bmjopen.bmj.com/site/about/guidelines.xhtml

Female	994 (58.8)
**Age (in years)**	
Below 5	185 (10.9)
5-9	263 (15.6)
10-19	453 (26.8)
20-29	318 (18.8)
30-39	213 (12.6)
40 -49	136 (8.0)
50-59	59 (3.5)
60 and above	64 (3.8)
**Level of education**	
Never attended	249 (14.7)
Primary	738 (43.6)
Secondary	614 (36.3)
Tertiary	90 (5.3)
**Occupation of the household head (N=357)**	
Formal employment	57 (16.0)
Unemployed	144 (40.3)
Informal employment	144 (40.3)
Student	2 (0.6)
Other	10 (2.8)

Household characteristics and risk factors

The characteristics of the 357 households that were included in the study are shown in table 2 below. With regard to source of drinking water, 33.3% used borehole water, 30% used piped communal water, 18.8%

For peer review only - http://bmjopen.bmj.com/site/about/guidelines.xhtml

used piped water from the yard and 12.3% used piped water from within the house and majority (68.3%) treated their drinking water. Of those who treated their drinking water, 54.9% added chlorine, 25.8% boiled water and added chlorine, 6.8% only boiled their water. Concerning use of toilets, only 16.2% used to flush toilets connected to the system, 18.2% used a flush toilet connected to a septic tank, 3.9% used ventilated pit latrines. Majority (98.3%) washed their hands after using the toilet and 91.4% washed their hands before eating (**Table 2**).

Table 2: Household characteristics and risk factors (n=357)

Variable	n (%)
**Source of drinking water**	
Borehole	119 (33.3)
Bottled water/Company for selling water	4 (1.1)
Piped water (in the yard)	67 (18.8)
Piped water (in the house)	44 (12.3)
Piped water (public)	107 (30.0)
Well (with a pump)	12 (3.4)
Well (unprotected)	3 (0.8)
Other	1 (0.3)
**Treatment of drinking water**	
No	113 (31.7)
Yes	244 (68.3)
**Methods used to treat water**	
Adding chlorine	134 (54.9)
Adding chlorine using water filter	1 (0.4)
Boil water	41 (16.8)
Boil water and adding chlorine	63 (25.8)
Boil water and using water filter	1 (0.4)
Letting water stand/stand	1 (0.4)
Using water filter	2 (0.8)
Other	1 (0.4)
**Use of flush toilet connected to system**	
Yes	58 (16.2)
No	299 (83.8)
**Use of flush toilet connected to septic tank**	
Yes	67 (18.2)
No	290 (81.2)
**Use of flush toilet connected to septic tank**	
Yes	11 (3.1)
No	346 (96.9)
**Use of pit latrines, ventilated or ameliorated**	
Yes	14 (3.9)

For peer review only - http://bmjopen.bmj.com/site/about/guidelines.xhtml

No	343 (96.1)
**Pit latrine with cement slab**	
Yes	168 (47.1)
No	189 (52.9)
**Pit latrine without cement slab**	
Yes	38 (10.6)
No	319 (89.4)
**No toilet (canal, open defecation, bush, field)**	
Yes	5 (1.4)
No	352 (98.6)
**Toilet facilities: others**	
Yes	3 (0.8)
No	354 (99.2)
**Toilet facilities shared with other households**	
Yes	182 (51.0)
No	175 (49.0)
**Washing of hands during the day**	
Yes	349 (97.8)
No	8 (2.2)
**Washing of hands after using the toilet**	
Yes	349 (98.3)
No	6 (1.7)
**Washing hands before eating**	
Yes	319 (91.4)
No	30 (8.6)
**Washing hands after eating (n=319 who answered yes above)**	
Yes	268 (76.8)
No	81 (23.2)
**Washing hands before cooking**	
Yes	194 (55.6)
No	155 (44.4)
**Washing hands after cleaning tables**	
Yes	79 (22.6%)
No	270 (77.4%)
**Washing hands after cleaning baby stool/diapers**	
Yes	113 (32.4)
No	236 (67.6)
**Washing hands after cleaning house**	
Yes	120 (34.4)
No	229 (65.6)
**Washing hands any time**	
Yes	67 (19.2)

For peer review only - http://bmjopen.bmj.com/site/about/guidelines.xhtml

No	282 (80.8)
**Washing hands any other time**	
Yes	4 (1.1)
No	345 (98.9)

Analysis of respondents who received the first OCV dose (n=1, 691)

Out of the total sample, 1, 375 (81.3%) received the first dose. Of these, 994 (58.8%) were female, 185 (10.9%) were aged between 1 and 4 years, 637 (37.7%) between 5 and 7 years, 543 (32.1%) between 18 and 35 years, 285 (16.9%) were aged between 36 and 65; 41 (2.4%) were aged above 65 years. The differences in characteristics between the respondents who received and those who did not receive the OCV during the first round are shown table 4 below (**Table 3**).

Table 3: Respondents who received the first OCV dose

First OCV Dose			
**Variable**	**Yes (n=1, 375)**	**No (n=316)**	**Total (n=1, 691)**
	**n(%)**	**n (%)**	
**Sex**			
Male	556 (40.4)	141 (20.2)	697 (41.2)
Female	819 (59.6)	175 (17.6)	994 (58.8
**Age (in years)**			
1-4	158 (11.5)	27 (8.5)	185 (10.9)
5-14	554(40.3)	83(26.3)	637 (37.7)
15-35	409 (29.7)	134 (42.4)	543 (32.1)
36-65	226 (16.4)	59 (18.7)	285 (16.9)
Above 65	28 (2.1)	13 (4.1)	41 (2.4)

Analysis of respondents who received the second OCV dose (n=1,691)

Out of the total sample of 1,691 that was included in the analysis for the second round of the OCV campaign, 1, 220 (72.1%) received the second dose. Of these, 744 (61.0%) were female, 143 (11.7%) were aged between 1 and 4 years, 520 (42.6%) were between 5 and 14 years, 338 (27.7%) were between 15 and 35 years, 189 (15.5%) were aged between 36 and 65; 30 (2.5%) were aged above 65 years (**table 5**).

Table 4: Respondents who received the second OCV dose

**Variable**	**Yes (n=1, 220)**	**No (n=471)**	**Total (n=1, 691)**

For peer review only - http://bmjopen.bmj.com/site/about/guidelines.xhtml

	n(%)	n (%)	
**Sex**			
Male	476 (39.0)	234 (49.6)	710 (42.0)
Female	744 (61.0)	237 (50.4)	981(58.0)
**Age**			
1-4	143 (11.7)	46 (9.8)	189 (11.1)
5-14	520 (42.6)	159 (33.8)	679 (40.2)
15-35	338 (27.7)	172 (36.5)	510 (30.2)
36-65	189 (15.5)	87 (18.4)	276 (16.3)
Above 65	30 (2.5)	7 (1.5)	37 (2.2)

For peer review only

Administrative coverage

The table below shows the target population and administrative coverage for the first and second OCV doses for each sub-district. The overall administrative coverage for the OCV first dose was 110%. Kanyama sub-district reported the highest coverage at 141.9%, followed by Chipata at 124.1%, Chawama at 98.4%; Matero sub-district had the lowest at 82.4%. The overall coverage for the second dose was 89%. Kanya sub-district recorded the highest coverage at 158%, followed by Chawama at 98%, Chipata at 63%. Matero recorded the lowest coverage at 62.9% (**Table 5**).

Table 5. Administrative coverage per sub-district

Sub-district	Target Population	Round 1		Round 2	
		Number (n)	Percentage (%)	Number (n)	Percentage
Kanyama	242, 302	343,760	141.9	382, 837	158
Chipata	368, 344	457,206	124.1	232, 057	63
Chawama	238, 804	235, 036	98.4	234, 028	98
Matero	356, 462	293,808	82.4	224, 340	62.9
**Total**	1, 205, 912	1, 329, 810	**110**	1,073, 262	**89**

OCV campaign coverage by dose based on the survey (n= 1691)

Table 6 below shows the OCV campaign coverage for each dose based on the survey results. The vaccine coverage for the first dose was 81.3%, (95%CI [79.24, 83.36]); the coverage for the second dose was 72.1% (95%CI [69.58, 74.62]); the two-dose coverage was 66% (95% CI [63.22, 68.78]). The drop-out rate was 18.8% (95%CI [14.51, 23.09]) (**Table 6**).

For peer review only - http://bmjopen.bmj.com/site/about/guidelines.xhtml

Table 6: OCV campaign coverage by dose

OCV dose	Number vaccinated	% (95% Confidence Interval)
First dose	1375	81.3 [79.24 – 83.36]
Second dose	1220	72.1 [69.58 –74.62]
Both doses	1116	66 [63.22 – 68.78]
Drop-out rate	318	18.8 [14.51 – 23.09]
At least one dose*	363	21.5 [17.27 – 25.73]

*Excludes those that received both doses

For peer review only

Multivariate logistic regression showing factors associated with the number of OCV doses received

The table below shows the factors associated with receiving the first and second doses of OCV. Compared to the male participants, female participants were 1.5 times more likely to receive the second dose (adjusted odds ratio,[AOR]=1.54 (95%CI [1.25, 1.91], p=0.00007). There was no association between sex and receiving the first OCV dose (AOR=1.19 [95%CI (0.93, 1.52], p=0.17). Compared to the children aged 1 to 4 years, those aged 15 to 35 (AOR=0.37, 95% CI(0.52[0.33, 0.82]; p=0.0043), 36 to 65 years (AOR=0.65, 95%CI[0.40, 1.08]; p=0.09) and those aged above 65 years (AOR=0.37, 95%CI [0.17, 0.80]; p=0.009) had less odds of receiving the first OCV dose. Similarly, the odds of receiving the second dose were significantly less among those aged 15 to 35 (AOR=0.63, 95%CI [0.43, 0.92]. p=0.017) and those aged 36 to 65 (AOR=0.699, 95%CI [0.46,1.062]; p=0.09). There was no significant difference in the odds of reciving the second dose between the those aged 1 to 4 and those aged above 65 (AOR=1.38, 95CI [0.57, 3.35]; p=o.48. For both the first and second doses, there was no significant difference in the odds of receiving the vaccine between those aged 1 to 4 and 5 to 14 years (AOR=1.14, 95% CI[0.71, 1.82]; p=0.58) and (AOR=1.05, 95%CI [0.72, 1.53], p=0.79), respectively. With regard to the household size, compared to households with four members, households with 5 to 7 members were more than two times more likely to receive the first dose (AOR=2.2, 95%CI[1.50, 3.40]; p<0.001). There was no significant association between the number of household members and receiving the second OCV dose (**Table 7).**

Table 7. Multivariate logistic regression: factors associated with the number of OCV doses received

		First dose versus o dose		Second dose versus 0 dose	
**Variable**	Category	**AOR [95% CI]**	P value	**AOR [95% CI]**	P-value
**Sex**					

For peer review only - http://bmjopen.bmj.com/site/about/guidelines.xhtml

	Male	1		1	
	Female	1.19 [0.93, 1.52]	0.17	1.54 [1.25, 1.91]	**0.00007**
**Age**					
	1-4	1		1	
	5-14	1.14[0.71,1.82]	0.58	1.05 [0.72, 1.53]	0.79
	15-35	0.52 [0.33, 0.82]	**0.0043**	0.63 [0.43, 0.92]	**0.017**
	36-65	0.65 [0.40, 1.08]	**0.09**	0.699 [0.46,1.062]	0.09
	Above 65	0.37 [0.17, 0.80]	**0.009**	1.38 [0.57, 3.35]	0.48
**Household size**	1 to 4	1		1	
	5-7	2.2 [1.50, 3.40]	**<0.001**	1 [0.6, 1.6]	0.85
	8-12	1.30 [0.50–3.50]	0.61	0.6 [0.2–2.2]	0.52
	12+	1.19 [0.92, 1.51]	0.27	0.54 [0.22, 1.91]	0.47

Discussion

The aim of this study was to determine the actual coverage (based on the population vaccinated) for the OCV campaign that was conducted in Lusaka district in 2018. Overall, our findings showed that the reported administrative coverage was much higher than the actual coverage. Coverage for the two-doses was 66.0%; coverage for the first and second dose was 81.3% and 72.1%, respectively. The drop- out rate was 18.8%. Sex, age and number household size were significantly associated with receiving the first and second doses. Compared to the male participants, female participants had higher odds of receiving the second dose. There was no significant difference in odds of receiving the first dose between the male and female participants. Older participants and elderly people were less likely to receive the first dose compared to the young children aged 1 to 4 years. Participants aged 15 to 35 years were significantly less likely to receive the second dose.

These findings show that the administrative coverage reported during the first and second rounds was much higher than the actual one from the population survey. These findings are consistent with a survey conducted by Semá and colleagues [26] in Mozambique which reported a difference between the OCV administrative coverage and the one from the population survey. According to these authors, the administrative coverage provided by the Mozambican MoH were 108.4% and 107.9% after the first and second rounds, respectively. However, based on the population survey, the overall vaccination coverage with at least one dose was 69.5% (95%CI: 51.2–88.2); the full dose coverage (two-dose) was 51.2% (95%CI: 37.9–64.3). These findings confirm that reliance on health facility data alone for calculating the OCV campaign coverage may mask low

For peer review only - http://bmjopen.bmj.com/site/about/guidelines.xhtml

coverage and prevent subsequent measures to improve programming. Thus, there is need for periodic validation of administrative coverage estimates with population-based methods [27].

Nevertheless, our findings corroborate previous studies that reported OCV campaign coverage using population survey data. For example, a survey conducted by Bwire and colleagues [28] on the use of surveys to evaluate an integrated oral cholera vaccine campaign in response to a cholera outbreak in Hoima district, Uganda, reported a (94% (95% CI 92% to 95%) coverage for at least one OCV dose and 78% (95% CI 76% to 81%) for two doses. Similarly, a Malawian survey by Sauvageot and colleagues [29] reported a high 2-dose coverage of 92.2% among participants who lived within 2km of lake Chilwa. In Iraq, Lam et al [30] reported an overall 2-dose OCV coverage of 87% in the targeted camps. Other studies have reported low coverage. A study conducted in Guinea by Luquero and others [31] reported a 2-dose coverage of 75.5%. In a Mozambican survey, Semá and colleagues reported an overall 2-dose coverage of 51.2% and 69.5% for at least one dose. Despite the variations in the reported campaign coverage, our findings corroborate previous studies and provide further evidence on the feasibility of conducting an OCV campaign during a cholera outbreak. In addition to traditional interventions, WHO guidelines require conducting OCV campaign during an outbreak.

Our findings show a vaccine drop-out rate of 18.8%. This findings is contrary to the WHO guidelines which recommend that, to be protected, an individual needs to receive two doses of Euvichol-Plus® [32]. The WHO guidelines also recommend that there must be a minimum of two weeks delay between two doses. Two doses of Euvichol-Plus® provide protection against cholera at least for three years, while one dose provides short term protection [33]. Similarly, Bekolo and colleagues [34] showed that patients who receive two doses of the OCV are 4.5 times less likely to develop severe disease than unvaccinated patients. Thus, individuals who receive only one dose do not get optimal protection from the vaccine. Future studies should focus on factors contributing to vaccine drop out after an individual has received the initial dose. Addressing these factors would increase coverage for both the first and second dose and maximize population benefits from the vaccine.

Finally, our findings show that sex, age and number household size were significantly associated with receiving the first and second doses of the vaccine. Female participants had higher odds of receiving the second dose than male counterparts. There was no significant difference in the odds of receiving the first dose between the male and female participants. The results also show significant differences in the odds of

For peer review only - http://bmjopen.bmj.com/site/about/guidelines.xhtml

receiving the vaccine among the age groups during both rounds; older participants (15 to 65 years and elderly (above 65 years) were less likely to receive the first dose compared to the children aged 1 to 4 years. Moreover, participants aged 15 to 35 years were significantly less likely to receive the second dose. These findings contradict those reported by Chitio and colleagues [35] which showed low vaccination coverage among Mozambican female respondents. However, our findings are consistent with those reported in a post OCV campaign survey by Semá and colleagues in Mozambique. These authors reported the highest coverage for the two-doses among adult females and lowest among adult males. These findings are also in line with those reported by Burnett and colleagues [36] in their study on the oral cholera vaccination coverage after the first global stockpile deployment in Haiti in 2014. These authors reported a higher percentage of children aged 5–14 years who received both recommended doses than did those ≥ 15 years. Public Health interventions planning OCV campaigns should take into consideration these sex and age differences in vaccine acceptability in order to achieve optimal vaccine coverage

Limitations

Potential limitations for this study should be acknowledged. First, although a random satellite-based sampling technique was used to select households, inclusion of all eligible participants found in a household into the study may have introduced selection bias. Second, our findings are based on a survey conducted following an OCV campaign in four urban sub-districts of Lusaka City. The generalizability of these findings to other settings within the country may be limited.

Despite these limitations, we believe this study has provided evidence on the actual coverage of the OCV campaign based on the survey and clearly shown that the reported administrative coverage was much higher than the actual one from the survey. These findings provide evidence on the importance of validating administrative vaccination campaign coverage with survey data based on the actual vaccinated population. Moreover, our findings confirm the feasibility of conducting an OCV campaign and achieving high coverage during a cholera outbreak in an urban setting like Lusaka city [37]. They corroborate previous campaigns and surveys conducted in other countries as well as the WHO guideline which recommend complementing traditional cholera control and preventions strategies (provision of safe drinking water, adequate sanitation, disease surveillance, health promotion and case management) with OCV campaign during an outbreak.

Conclusion

For peer review only - http://bmjopen.bmj.com/site/about/guidelines.xhtml

Our findings confirm the 2018 OCV campaign coverage and show that the reported administrative coverage was much higher than the actual one based on population that was vaccinated. These findings highlight the need for follow up surveys to validate administrative coverage estimates using population-based methods. Reliance on health facility data alone may mask low coverage and prevent measures to improve programming. Our findings highlight important factors associated with vaccine acceptance: sex, age and household size. Public health interventions planning OCV campaigns should take into consideration the sex, age and other sociodemographic differences in order to achieve optimal vaccine coverage. These findings also confirm the feasibility of conducting an OCV campaign during a cholera outbreak in an urban setting and highlight the importance of complementing traditional cholera control and prevention interventions with an OCV campaign to improve health outcomes in a cholera outbreak. Future intervention and studies should focus on: 1) engaging male adults and the aged population; 2) determining the long term effectiveness of single and two-doses in the population as well as their cost-effectiveness.

Author contribution

All authors contributed substantially to the development of this manuscript. VM, CS, NK, BM, OC ANM, WN, MK, LM, RH, HM and NS contributed to the conception of the study. NK, BM, OC, NS, HM and LC supervised the data collection process. WN, CS and RH conducted the data analysis. CS, HM, OC and BM drafted the manuscript; BM and VM edited it; CS revised the manuscript. All authors read, commented on and approved the final manuscript. CS is the guarantor for the manuscript.

Funding Statement

Ministry of Health and World Health Organization (award/grant number: N/A)

Competing Interests

All authors declare that they have no conflicting interests in this work

Data Sharing

For peer review only - http://bmjopen.bmj.com/site/about/guidelines.xhtml

Data are available upon reasonable request from the corresponding author and with permission of the ERES ethics review board.

Costs and payments

There was no cash payment provided to participants for any portion of the study. Participants volunteered their time taken to participate in the study. As a small token of appreciation for their time and opportunity costs, participants received a bottle of water or soft drink, in line with the ERES guideline.

Acknowledgements

We thank Lusaka District Health Management Team who provided field research assistants and study participants. The GTFCC supported the country with OCV stocks and operations funds.

Ethics approval

Ethical approval for the study was obtained from the Excellence in Research Ethics and Science (ERES, 2019-09-001) Converge Committee and authority to conduct the study was obtained from the National Health Research Authority. Written informed consent was obtained from all participants in the study.

References

1. Mukhopadhyay A.K., Takeda Y., Balakrish Nair. Cholera Outbreaks in the El Tor Biotype Era and the Impact of the New El Tor Variants. In: Nair, G., Takeda, Y. (eds) Cholera Outbreaks. Current Topics in Microbiology and Immunology, 2014; vol 379. Springer, Berlin, Heidelberg. https://doi.org/10.1007/82_2014_363
2. Son MS, Megli CJ, Kovacikova G, Qadri F, Taylor RK. Characterization of Vibrio cholerae O1 El Tor biotype variant clinical isolates from Bangladesh and Haiti, including a molecular genetic analysis of virulence genes. J Clin Microbiol; 2011;49(11):3739-49. doi: 10.1128/JCM.01286-11.
3. Deen J, Mengel MA, Clemens JD. Epidemiology of cholera. Vaccine, 2020;38 Suppl 1:A31-A40. doi: 10.1016/j.vaccine.2019.07.078

For peer review only - http://bmjopen.bmj.com/site/about/guidelines.xhtml

4. Rosewell A, Addy B, Komnapi L, Makanda F, Ropa B, Posanai E et al. Cholera risk factors, Papua New Guinea, 2010. BMC Infect Dis, 2012; 12:287. doi: 10.1186/1471-2334-12-287
5. WHO, 2022. Cholera. Available at: https://www.who.int/news-room/fact-sheets/detail/cholera, accessed on 10th July, 2022
6. CDC, 2022. About Global Cholera. Available at: About Global Cholera (cdc.gov), accessed on 1st December, 2022
7. CDC, 2022. Data and Statistics on Global Cholera. Available at: Data and Statistics on Global Cholera (cdc.gov),
8. WHO, 2011. WHO Consultation on oral cholera vaccine (OCV) stockpile strategic framework: potential objectives and possible policy options. Available on the Internet at: www.who.int/vaccines-documents
9. Lopez AL, Gonzales ML, Aldaba JG, Nair GB. Killed oral cholera vaccines: history, development and implementation challenges. Ther Adv Vaccines. 2014 Sep;2(5):123-36. doi: 10.1177/2051013614537819. PMID: 25177492; PMCID: PMC4144262
10. WHA. Stop Cholera. Available at: The 64th World Health Assembly Resolution for Renewed Efforts to Prevent and Control Cholera (May 2011) | DOVE: Stop Cholera
11. WHO. Cholera vaccine. Available at: Immunization, Vaccines and Biologicals (who.int)
12. Bi Q, Ferreras E, Pezzoli L, Legros D, Ivers LC, Date K, et al. Oral Cholera Vaccine Working Group of The Global Task Force on Cholera Control. Protection against cholera from killed whole-cell oral cholera vaccines: a systematic review and meta-analysis. Lancet Infect Dis. 2017; 10:1080-1088. doi: 10.1016/S1473-3099(17)30359-6.
13. Franke MF, Ternier R, Jerome JG, Matias WR, Harris JB, Ivers LC. Long-term effectiveness of one and two doses of a killed, bivalent, whole-cell oral cholera vaccine in Haiti: an extended case-control study. Lancet Glob Health. 2018 Sep;6(9):e1028-e1035. doi: 10.1016/S2214-109X(18)30284-5.
14. Franke MF, Jerome JG, Matias WR et al. Comparison of two control groups for estimation of oral cholera vaccine effectiveness using a case-control study design. *Vaccine.* 2017; 35: 5819-582
15. Wierzba TF, Kar SK, Mogasale VV et al. Effectiveness of an oral cholera vaccine campaign to prevent clinically-significant cholera in Odisha State, India. *Vaccine.* 2015; 33: 2463-2469
16. Sialubanje C, Kapina M, Chewe O*, et al.* Effectiveness of two doses of Euvichol-plus oral cholera vaccine in response to the 2017/2018 outbreak: a matched case–control study in Lusaka, Zambia

For peer review only - http://bmjopen.bmj.com/site/about/guidelines.xhtml

BMJ Open 2022;12:e066945. doi: 10.1136/bmjopen-2022-06694

17. Kanungo S, Sen B, Ramamurthy T, Sur D, Manna B, Pazhani GP et al. Safety and immunogenicity of a live oral recombinant cholera vaccine VA1.4: a randomized, placebo controlled trial in healthy adults in a cholera endemic area in Kolkata, India. PLoS One, 2014; 1;9(7):e99381. doi: 10.1371/journal.pone.0099381.
18. Baik YO, Choi SK, Olveda RM, Espos RA, Ligsay AD, Montellano MB et al. A randomized, non-inferiority trial comparing two bivalent killed, whole cell, oral cholera vaccines (Euvichol vs Shanchol) in the Philippines. Vaccine, 2015; 17;33(46):6360-5. doi: 10.1016/j.vaccine.2015.08.075
19. Poncin M, Zulu G, Voute C, Ferreras E, Muleya MC, Malama K et al. Implementation research: reactive mass vaccination with single-dose oral cholera vaccine, Zambia. Bull World Health Organ, 2018;96:86–93 | doi: http://dx.doi.org/10.2471/BLT.16.18924

20. WHO, 2017. African Region. Available at: Disease outbreak - Cholera - Zambia - 11 December 2017 | WHO | Regional Office for Africa,
21. WHO 2021. Mass Cholera Vaccination Campaign Launched. Available at: Mass cholera vaccination campaign launched (who.int).
22. Sialubanje C, Kapina M, Chewe O, *et al.* Effectiveness of two doses of Euvichol-plus oral cholera vaccine in response to the 2017/2018 outbreak: a matched case–control study in Lusaka, Zambia. *BMJ Open* 2022;12:e066945. doi: 10.1136/bmjopen-2022-066945
23. Sialubanje C, Mukumbuta N, Ng'andu M, et al. Perspectives on the COVID-19 vaccine uptake: a qualitative study of community members and health workers in Zambia. BMJ Open 2022;12:e058028. doi: 10.1136/bmjopen-2021-058028
24. Sialubanje C, Sitali DC, Mukumbuta N, *et al.* Perspectives on factors influencing transmission of COVID-19 in Zambia: a qualitative study of health workers and community members. *BMJ Open* 2022;12:e057589. doi: 10.1136/bmjopen-2021-057589
25. Central Statistics Office, Ministry of Health and ICF International. Zambia Demographic and Health Survey 2013-14. Rockville, Maryland, USA; 2014.
26. Semá Baltazar SC, Rafael F, Langa JPM, Chicumbe S, Cavailler P, Gessner BD, et al. Oral cholera vaccine coverage during a preventive door-to-door mass vaccination campaign in Nampula, Mozambique. PLoS One. 2018 Oct 3;13(10):e0198592. doi: 10.1371/journal.pone.0198592.

For peer review only - http://bmjopen.bmj.com/site/about/guidelines.xhtml

27. Janmohamed A, Doledec D. Comparison of administrative and survey data for estimating vitamin A supplementation and deworming coverage of children under five years of age in Sub-Saharan Africa. Trop Med Int Health. 2017;22(7):822-829. doi: 10.1111/tmi.12883. Epub 2017 May 26. PMID: 28449319

28. Bwire G, Roskosky M, Ballard A, Brooks WA, Okello A, Rafael F et al. Use of surveys to evaluate an integrated oral cholera vaccine campaign in response to a cholera outbreak in Hoima district, Uganda. BMJ Open. 2020 Dec 10;10(12):e038464. doi: 10.1136/bmjopen-2020-038464.

29. Sauvageot D, Saussier C, Gobeze A, Chipeta S, Mhango I, Kawalazira G, et al. Oral cholera vaccine coverage in hard-to-reach fishermen communities after two mass Campaigns, Malawi, 2016. Vaccine,2017; 12;35(38):5194-5200. doi: 10.1016/j.vaccine.2017.07.104. Epub 2017 Aug 10. PMID: 28803712.

30. Lam E, Al-Tamimi W, Russell SP, Butt MO, Blanton C, Musani AS, Date K. Oral Cholera Vaccine Coverage during an Outbreak and Humanitarian Crisis, Iraq, 2015. Emerg Infect Dis. 2017;23(1):38-45. doi: 10.3201/eid2301.160881.

31. Luquero FJ, Grout L, Ciglenecki I, Sakoba K, Traore B, Heile M et al. First outbreak response using an oral cholera vaccine in Africa: vaccine coverage, acceptability and surveillance of adverse events, Guinea, 2012. PLoS Negl Trop Dis. 2013;7(10):e2465. doi: 10.1371/journal.pntd.0002465.

32. WHO, 2022. Preventing and controlling cholera in endemic countries. Available at: Preventing and controlling cholera in endemic countries (who.int),

33. Desai SN, Pezzoli L, Martin S, Costa A, Rodriguez C, Legros D, Perea W. A second affordable oral cholera vaccine: implications for the global vaccine stockpile. Lancet Glob Health. 2016 Apr;4(4):e223-4. doi: 10.1016/S2214-109X(16)00037-1. PMID: 27013303

34. Bekolo CE, JAF van Loenhout, Rodriguez-Llanes, JM, Rumunu J, Ramadanc OP & Guha-Sapir D. A retrospective analysis of oral cholera vaccine use, disease severity and deaths during an outbreak in South Sudan. Bull World Health Organ 2016;94:667–674 | doi: http://dx.doi.org/10.2471/BLT.15.166892

35. Elias Chitio JJ, Baltazar CS, Langa JP, et al. Pre-emptive oral cholera vaccine (OCV) mass vaccination campaign in Cuamba District, Niassa Province, Mozambique: feasibility, vaccination coverage and delivery costs using CholTool BMJ Open 2022;**12:**e053585. doi: 10.1136/bmjopen-2021-053585

36. Burnett EM, Francois J, Sreenivasan N, Wannemuehler K, Faye PC, Tohme RA et al. Oral cholera vaccination coverage after the first global stockpile deployment in Haiti, 2014. Vaccine. 2019 Oct 8;37(43):6348-6355. doi: 10.1016/j.vaccine.2019.09.018. Epub 2019 Sep 11. PMID: 31521413.

37. Vogel L. Mass cholera vaccination finally possible. CMAJ.2015; 187(7):E207-E208. doi: 10.1503/cmaj.109-5021.

For peer review only

Figure 1: Recruitment algorithm

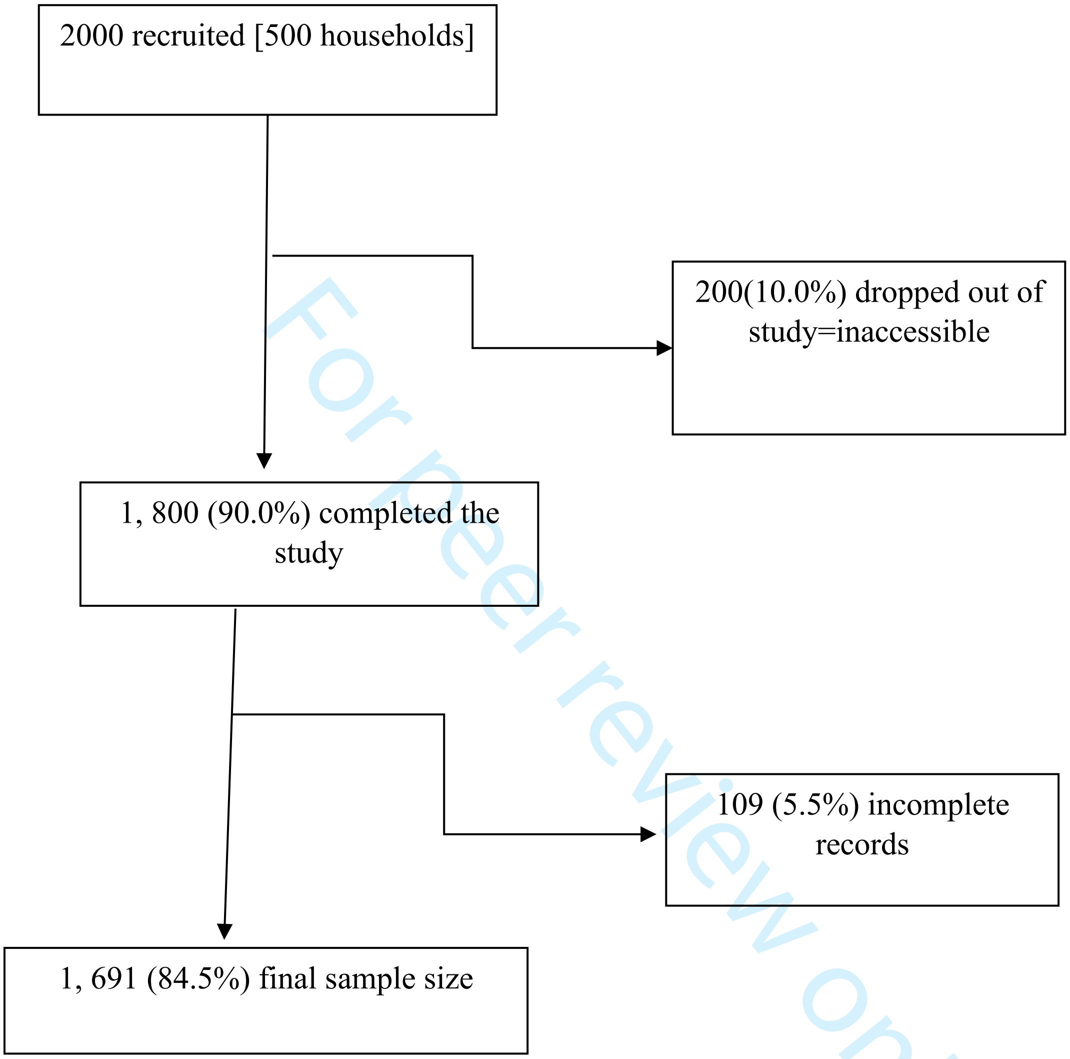

For peer review only

STROBE Statement—Checklist of items that should be included in reports of Descriptive Studies

Item	Recommendation	Page
Title and abstract	Indicate the study's design with a commonly used term in the title or the abstract	1
	Provide in the abstract an informative and balanced summary of what was done and what was found	2
Introduction		
Background/rationale	Explain the scientific background and rationale for the investigation being reported	4
Objectives	State specific objectives, including any pre-specified hypotheses	5
Methods		
Study design	Present key elements of study design early in the paper	5
Setting and population	Describe the setting, locations, and relevant dates, including periods of recruitment, exposure, follow-up, and data collection	6
Study size	Explain how the study size was arrived at	6
Data collection	For each variable of interest, give sources of data and details of methods of assessment (measurement). Describe comparability of assessment methods if there is more than one group	6
Quality assurance	Describe any efforts to address potential sources of bias	8
Statistical methods	Describe all statistical methods, including those used to control for confounding	8
	If applicable, explain how loss to follow-up was addressed	
Patient and public involvement		9
Ethical considerations		9
Results		
Descriptive statistics	(a) Give characteristics of study participants (eg demographic, clinical, social) and information on exposures and potential confounders<br>Descriptive data<br>(b) Indicate number of participants with missing data for each variable of interest	10
Main results		13
Discussion		15
Limitations		16
Conclusion		17
References		18

For peer review only

BMJ Open

Euvichol-Plus Vaccine Campaign Coverage during the 2017/2018 Cholera outbreak in Lusaka District, Zambia: A Cross Sectional Descriptive Study

Journal:	*BMJ Open*
Manuscript ID	bmjopen-2022-070796.R1
Article Type:	Original research
Date Submitted by the Author:	13-Aug-2023
Complete List of Authors:	Mukonka, Victor M; Levy Mwanawasa Medical University, School of Public Health; The Copperbelt University, School of Medicine<br>Sialubanje, Cephas; Levy Mwanawasa Medical University, School of Public Health<br>Matapo, Belem; World Health Organization, Disease Surveillance<br>Chewe, Orbrie; Zambia National Public Health Institute, Surveillance and Disease Intelligence ; Zambia Ministry of Health, Public Health<br>Ngomah, Albertina; Zambia National Public Health Institute, Communication Information & Research<br>Ngosa, Willaim; Zambia National Public Health Institute, Communication Information and Research<br>Hamoonga, Raymond; Zambia National Public Health Institute, Surveillance and Disease Intelligence<br>Sinyange, Nyambe; Zambia National Public Health Institute, Surveillance and Disease Intelligence<br>Mzyece, Hannah; Zambia National Public Health Institute, Surveillance and Disease Intelligence<br>Mazyanga, Lucy; Zambia National Public Health Institute, Communication Information and Research,<br>Bakyaita, Nathan; World Health Organization<br>Kapata, Nathan; Zambia National Public Health Institute, 4Epidemic Preparedness and Response, Zambia National Public Health Institute, Lusaka, Zambia
**Primary Subject Heading**:	Epidemiology
Secondary Subject Heading:	Public health, Infectious diseases, Health services research
Keywords:	EPIDEMIOLOGY, EPIDEMIOLOGIC STUDIES, INFECTIOUS DISEASES, Tropical medicine < INFECTIOUS DISEASES, PUBLIC HEALTH

SCHOLARONE™
Manuscripts

I, the Submitting Author has the right to grant and does grant on behalf of all authors of the Work (as defined in the below author licence), an exclusive licence and/or a non-exclusive licence for contributions from authors who are: i) UK Crown employees; ii) where BMJ has agreed a CC-BY licence shall apply, and/or iii) in accordance with the terms applicable for US Federal Government officers or employees acting as part of their official duties; on a worldwide, perpetual, irrevocable, royalty-free basis to BMJ Publishing Group Ltd ("BMJ") its licensees and where the relevant Journal is co-owned by BMJ to the co-owners of the Journal, to publish the Work in this journal and any other BMJ products and to exploit all rights, as set out in our licence.

The Submitting Author accepts and understands that any supply made under these terms is made by BMJ to the Submitting Author unless you are acting as an employee on behalf of your employer or a postgraduate student of an affiliated institution which is paying any applicable article publishing charge ("APC") for Open Access articles. Where the Submitting Author wishes to make the Work available on an Open Access basis (and intends to pay the relevant APC), the terms of reuse of such Open Access shall be governed by a Creative Commons licence – details of these licences and which Creative Commons licence will apply to this Work are set out in our licence referred to above.

Other than as permitted in any relevant BMJ Author's Self Archiving Policies, I confirm this Work has not been accepted for publication elsewhere, is not being considered for publication elsewhere and does not duplicate material already published. I confirm all authors consent to publication of this Work and authorise the granting of this licence.

For peer review only

Euvichol-Plus Vaccine Campaign Coverage during the 2017/2018 Cholera outbreak in Lusaka District, Zambia: A Cross Sectional Descriptive Study

Victor Mukonka[1,2,3]; Cephas Sialubanje[1], Belem Matapo[5]; Orbrie Chewe[3,6]; Albertina Moraes Ngomah[7]; Willaim Ngosa[7], Raymond Hamoonga[3], Nyambe Sinyange[3], Hannah Mzyece[3], Lucy Mazyanga[7], Nathan Bakyaita[5]; Nathan kapata[4]

[1]School of Public Health, Levy Mwanawasa Medical University, Lusaka, Zambia

[2]School of Medicine, Copperbelt University, Kitwe, Zambia

[3]Surveillance and Disease Intelligence, Zambia National Public Health Institute, Lusaka, Zambia

[4]Epidemic Preparedness and Response, Zambia National Public Health Institute, Lusaka, Zambia

[4]Department of Public Health, Ministry of Health, Lusaka, Zambia

[5]Disease Surveillance, World Health Organisation, Lusaka, Zambia

[6]Department of Public Health, Ministry of Health, Lusaka, Zambia

[7]Communication Information and Research, Zambia National Public Health Institute, Lusaka, Zambia

[8]Laboratory System and Network, Zambia National Public Health Institute, Lusaka, Zambia

Corresponding Author: Cephas Sialubanje

csialubanje@yahoo.com

For peer review only - http://bmjopen.bmj.com/site/about/guidelines.xhtml

Abstract

Objective: To determine the coverage for the oral cholera vaccine (OCV) campaign conducted during the 2017/18 cholera outbreak in Lusaka, Zambia.

Study design: A descriptive cross-sectional study employing survey method conducted among 1,691 respondents from 369 households following the second round of the 2018 OCV campaign

Study Setting: Four primary health care facilities and their catchment areas in Lusaka city (Kanyama, Chawama, Chipata and Matero sub-districts)

Participants: A total of 1,691 respondents 12 months and older sampled from 369 households where the campaign was conducted. A satellite-map based sampling technique was used to randomly select households.

Data management and analysis: A pre-tested electronic questionnaire uploaded on an electronic tablet (ODK Version 1.12.2) was used for data collection.: Descriptive statistics were computed to summarise respondents' characteristics and OCV coverage per dose. Bivariate analysis (Chi-square test) was conducted to stratify OCV coverage according to age and sex for each round ($p<0.05$).

Results

The overall coverage for the first, second and two-doses were 81.3% (95%CI [79.24, 83.36]), 72.1% (95%CI [69.58, 74.62]), and 66% (95% CI [63.22, 68.78], respectively. The drop-out rate was 18.8% (95%CI [14.51, 23.09]). Of the 81.3% who received the first dose, 58.8% were female. Among those who received the second dose majority (61.0%) were female aged between 5 and 14 years (42.6%) and 15 35 years (27.7%). Only 15.5% of the participants aged between 36 and 65 and 2.5% among those aged above 65 years received the second dose.

Conclusion

These findings confirm the 2018 OCV campaign coverage and highlight the need for follow up surveys to validate administrative coverage estimates using population-based methods. Reliance on health facility data alone may mask low coverage and prevent measures to improve programming. Future public health interventions should consider sociodemographic factors in order to achieve optimal vaccine coverage.

Word count: 295

For peer review only - http://bmjopen.bmj.com/site/about/guidelines.xhtml

Strengths and weakness

- Conducting the survey shortly (within a month) after the second round of the OCV campaign minimized recall bias
- Verification of the self-reported vaccination status with the vaccination certificate card increased internal validity of the findings
- Use of a satellite-map based sampling technique to randomly select households reduced selection bias.
- Use of a pre-tested electronic questionnaire enhanced data quality and ensured internal validity of the study
- Although a random satellite-based sampling technique was used to select households, inclusion of all eligible participants found in a household into the study may have introduced selection bias.

For peer review only

For peer review only - http://bmjopen.bmj.com/site/about/guidelines.xhtml

1.0 Introduction

1.1. Background

Cholera is a rapidly dehydrating diarrheal illness caused by the toxigenic bacterium *Vibrio cholerae* serogroups O1 and O139 [1]. The 01 serogroup has two biotypes, classical and E1 Tor [2]. During the past years, cholera has spread from the Indian subcontinent to involve nearly the whole world and cause pandemics in Africa and South America. Since 1817, the world has experienced seven cholera pandemics, the first six of which were caused by the classical O1 biotype. The current (seventh) pandemic, which began in 1961 and largely confined to Asia and Africa, is mainly caused by the El Tor O1 biotype which has essentially displaced the classical biotype globally since 1993 [2,3]. Over-crowding, poverty, poor sanitation facilities, lack of access to clean and safe water and food contamination increase the risk for cholera outbreaks [4]. Global annual estimates of cholera range from 1.3 to 4. Million cases and 21,000 to 143,000 deaths [5-7]. However, the actual burden of the disease is unknown due to poor reporting, limited surveillance, and lack of laboratory capacity. In Africa, the burden of cholera is mainly in sub-Sahara Africa with approximately 19 countries more affected by epidemics, with 105, 287 cases and 1882 deaths [5-7].

Although access to and provision of safe drinking water, adequate sanitation, disease surveillance, health promotion and case management remain the mainstay of cholera prevention and control [8], use of reasonably priced, safe and effective vaccines can provide an additional intervention for cholera prevention and control [9]. The 2011 World Health Assembly (WHA) [10] resolution 64.15 called for implementation of an integrated and comprehensive approach to cholera control. Consequently, in 2011 the World Health Organization (WHO) established a global stockpile under the supervision of the International Coordination Group on Vaccine Provision to strengthen the capacity for action against cholera in emergency settings and recommended use of oral cholera vaccines (OCV) in endemic and epidemic settings [11]. Subsequently, four killed, whole-cell OCVs–Dukoral, mORCVAX, Shanchol and Euvichol–are currently available and licensed for use in different countries [11]. Shanchol and Euvichol are killed, whole-cell, bivalent O1 and O139 and licensed for use in children ≥ 1 year and require 2 doses given 2 weeks apart. They are the preferred vaccines for public health use as they do not need a buffer [12].

Numerous studies have demonstrated the efficacy, safety and immunogenicity of the killed whole-cell OCV [13-15]. For example, a randomized placebo-controlled safety and immunogenicity study conducted among healthy adults in cholera endemic area in Kolkata, India [16] found that the reformulated oral killed whole cell

For peer review only - http://bmjopen.bmj.com/site/about/guidelines.xhtml

vaccine was safe, well tolerated and highly immunogenic. Studies have also shown that Euvichol has the same formulation as Shanchol and that it has non-inferior sero-conversion rates to those of Shanchol [17.18]. The study also reported a fourfold rise in serum *v.cholera* 01 vibriocidal antibody titers among 53% adults and 80% children.

Following the 2017/18 cholera outbreaks [19] and based on lessons from the previous outbreaks [20], the Zambian Ministry of Health (MoH) through WHO and with support from the Global Task Force on Cholera Control (GFTCC), procured 2,070,100 vaccine doses of the Euvichol-Plus™ OCV campaign to support the short- to- medium term interventions to control cholera. The first OCV round was launched on the 10th January 2018 in the four affected sub-districts of Lusaka (that is, Chawama, Matero, Kanyama and Chipata). Due to logistical challenges, and while waiting for delivery of the complete vaccine stocks of OCV doses, the second round of the campaign was implemented in two phases. Phase 1 was conducted between 5 and 14 February 2018 and covered two of the four subdistricts (Chawama and Kanyama); phase 2 was implemented from 18 to 25 April 2018 and included the other two subdistricts (Chipata and Matero). In addition to the OCV campaign, the Zambian MOH implemented the following interventions: provision of safe water, chlorination of unsafe water sources, improved sanitation and solid waste management, training of community workers, cleaning of the environment with community involvement and health promotion campaigns through door-to-door campaigns, public address system and mass media (radio and television).

1.2. Objectives

The aim of this study was to determine the actual OCV coverage during the 2018 OCV campaign conducted in Lusaka district. Information on the actual vaccination coverage (based on the population vaccinated) is required to validate the administrative coverage that was reported at 109% for the first round. Currently, there is a lack of evidence on the coverage of OCV campaigns among the Zambian population.

2. Methods

2.1. Study design

This was a survey conducted among 1,691 respondents from 369 households within a month following the second round of the 2018 OCV campaign in the four subdistricts of Lusaka, Zambia.

2.2. Study site and participants

For peer review only - http://bmjopen.bmj.com/site/about/guidelines.xhtml

The study sites included the four sub-districts of Lusaka (Kanyama, Chawama, Chipata and Matero) where the 2018 OCV campaign was conducted. The combined population for the four subdistricts was 1 543 507. (see our previous work on the 2018 Lusaka cholera outbreak and for the details on the study setting [21-23]. The study population included all individuals older than 12 months residing in the study sites.

2.3. Inclusion criteria

To be eligible to participate in the survey, respondents needed to:

a) Be residing in study sites for at least two weeks prior to the last date of vaccine administration.
b) Older than 12 months
c) Provide consent to participate in the study

2.4. Sample size determination

To estimate the sample size for the survey, the following assumptions were made: a) 50% of the population received two doses; b) confidence interval of 95% (equivalent to an alpha error of 5%); precision of 5; c) design effect of 3; and d) 15% missing data and non-response (18,19). This gave an estimated sample size of 1, 325 of respondents. To estimate the required number of households, the Zambia Demographic and Health Survey report of 2013-14 [24] which showed a mean number of 4 persons per household was used. This gave a total of 332 households (1325/4=331.25) to be included in the study. A household was defined as a group of people sleeping under the same roof and sharing meals every day for at least two previous weeks.

Sampling techniques

A random satellite-based sampling technique was used to select households for inclusion in the study. First, a random point was generated in the sampling area and retained if it coincided with a household (+/- 10 meters error); the process was repeated until the required sample size was reached. All eligible individuals (ranging from 1 to 6 participants per household) in the selected household at the time of the survey were selected.

2.5. Data Collection

For peer review only - http://bmjopen.bmj.com/site/about/guidelines.xhtml

Twenty four (24) trained and experienced research assistants were recruited from the previously conducted coverage survey in Lusaka district. Research assistants were trained for four days; the training comprised classroom-based theory for 3 three days followed by field work for one day. Topics covered during classroom training included the a) purpose of the study, b) study methods including sampling techniques; and d) electronic questionnaire administration. Field work included testing the data collection tools, household location using geographical positioning system (GPS) technique. Data collection instruments were revised based on feedback from the research assistants.

Research assistants worked in teams of four, comprising three members and a team leader. Research assistants administered the questionnaire and read out the interview questions during the face-to-face interview which were conducted in the local language. In addition, the research assistants collected information on the number and proportion of people that received the vaccine in the household; collected GPS coordinates and data on health promotion and cholera prevention. If responders were not found at the house, a second visit was organized later in the day or on a second day. The household was skipped if respondents were not found or refused to participate during the second visit.

2.6. Data Collection Instruments

A pre-tested electronic questionnaire uploaded on the electronic tablet using ODK Version 1.12.2 was used to collect data during face-to-face interviews. The questionnaire comprised questions on eligibility, socio-demographic data (age, sex and household size), vaccination status (self-reported and card-confirmed) and acceptability data (perception and beliefs about the vaccine, vaccination adverse events). To ensure quality in the data collection process, the team leader cross checked the completed questionnaires on a daily basis. Completed and verified questionnaires were transmitted to the server at Zambia National Public Health Institute.

2.7. Quality assurance

To ensure quality assurance in the data collection process, the following measures were taken: a) the survey was conducted about 2 weeks after completion of the second round of the vaccination campaign to minimise recall bias; b) research assistants were trained on data collection methods; c) an electronic data collection tool uploaded on the ODL kit was pre-tested and revised accordingly; c) field supervisors worked with the research assistants and verified the data entry before the data was transmitted to the sever; d) research

For peer review only - http://bmjopen.bmj.com/site/about/guidelines.xhtml

assistants had no access to the transmitted data; e) householders who were not present during the field were revisited to avoid selection bias.

2.8. Data Processing and Analysis

Data was retrieved from the server, cleaned and saved on an excel sheet. Analysis was done using STATA version 12.4. Descriptive statistics were computed to summarise respondent and household characteristics and OCV campaign coverage for each round. Vaccination status was determined using both the vaccination certificate (card-confirmed) and self-reports. In addition, Chi-square test was used to compute the OCV vaccine coverage by sex and age group for each round (95% Confidence Interval, p<0.005). To estimate coverage of the 2018 vaccination campaign, the following indicators were computed: a) vaccine coverage for two doses, b) vaccine coverage for the first round; c) vaccine coverage for the second round; d) vaccine coverage with at least one dose. Vaccine coverage was calculated by dividing the number of individuals reporting being vaccinated by the survey population and expressed as a percentage. In addition, the drop-out rate the two rounds was calculated as shown below:

$$Drop\ out\ rate = \left(\frac{Vaccin\_1 - Vaccin\_1\&\_2}{Vaccin\_1} \times 100\right)$$

Vaccin_1 = the number of people vaccinated during the 1st round.

Vaccin_1& 2 = the number of people vaccinated during the 1st and the 2nd round.

2.9. Patient and Public Involvement

The study design was determined by the research team. Participants and the public were not directly involved in the conceptualisation and design of the study. However, selection of the study sites was done in consultation with stakeholders from the Ministry of Health and Zambia National Public Health Institute. Selection of study participants was done in collaboration with the provincial and district health managers. A dissemination meeting was held in Lusaka and study findings shared with key stakeholders, including the WHO, MoH and community leaders in the health facilities where the study was conducted. A final report was also written and shared with the funding organization.

3. Results

For peer review only - http://bmjopen.bmj.com/site/about/guidelines.xhtml

Participants

A total of 2,000 participants were recruited into the study. Of these, 200 (10%) could not be traced due to access challenges in the urban slums and thus dropped out of the study. A total of 1800 (90%) participants completed the study, out of whom 109 (5.5%) had incomplete records and were not included in the final analysis. A total final sample of 1, 691 respondents (84.5%) were included in the final analysis. A summary of the recruitment algorithm of study participants is shown in figure 1 (**Supplementary figure 1**)

Demographic characteristics.

Most respondents were female (58.8%) and aged below 30 (53.3%). Slightly more than two fifths (43.6%) of the respondents had completed primary education, 36.3% had completed secondary education and 5.3% had attended tertiary education and 14.7% never attended school. With regard to occupation, 16.0% were in formal employment, 40.3% were in informal employment and 40.3% were unemployed (**Table 1**).

Table 1: Demographic characteristics

**Variable**	**n (%)**
**Sex**	
Male	697 (41.2)
Female	994 (58.8)
**Age (in years)**	
Below 5	185 (10.9)
5-9	263 (15.6)
10-19	453 (26.8)
20-29	318 (18.8)
30-39	213 (12.6)
40 -49	136 (8.0)
50-59	59 (3.5)
60 and above	64 (3.8)

For peer review only - http://bmjopen.bmj.com/site/about/guidelines.xhtml

Level of education	
Never attended	249 (14.7)
Primary	738 (43.6)
Secondary	614 (36.3)
Tertiary	90 (5.3)
**Occupation of the household head (N=357)**	
Formal employment	57 (16.0)
Unemployed	144 (40.3)
Informal employment	144 (40.3)
Student	2 (0.6)
Other	10 (2.8)

For peer review only

Household characteristics and risk factors

The characteristics of the 357 households that were included in the study are shown in table 2 below. With regard to source of drinking water, 33.3% used borehole water, 30% used piped communal water, 18.8% used piped water from the yard and 12.3% used piped water from within the house and majority (68.3%) treated their drinking water. Of those who treated their drinking water, 54.9% added chlorine, 25.8% boiled water and added chlorine, 6.8% only boiled their water. Concerning use of toilets, only 16.2% used to flush toilets connected to the system, 18.2% used a flush toilet connected to a septic tank, 3.9% used ventilated pit latrines. Majority (98.3%) washed their hands after using the toilet and 91.4% washed their hands before eating (**Table 2**).

Table 2: Household characteristics and risk factors (n=357)

Variable	n (%)
**Source of drinking water**	
Borehole	119 (33.3)
Bottled water/Company for selling water	4 (1.1)
Piped water (in the yard)	67 (18.8)
Piped water (in the house)	44 (12.3)

For peer review only - http://bmjopen.bmj.com/site/about/guidelines.xhtml

Piped water (public)	107 (30.0)
Well (with a pump)	12 (3.4)
Well (unprotected)	3 (0.8)
Other	1 (0.3)
**Treatment of drinking water**	
No	113 (31.7)
Yes	244 (68.3)
**Methods used to treat water**	
Adding chlorine	134 (54.9)
Adding chlorine using water filter	1 (0.4)
Boil water	41 (16.8)
Boil water and adding chlorine	63 (25.8)
Boil water and using water filter	1 (0.4)
Letting water stand/stand	1 (0.4)
Using water filter	2 (0.8)
Other	1 (0.4)
**Use of flush toilet connected to system**	
Yes	58 (16.2)
No	299 (83.8)
**Use of flush toilet connected to septic tank**	
Yes	67 (18.2)
No	290 (81.2)
**Use of flush toilet connected to septic tank**	
Yes	11 (3.1)
No	346 (96.9)
**Use of pit latrines, ventilated or ameliorated**	
Yes	14 (3.9)
No	343 (96.1)
**Pit latrine with cement slab**	
Yes	168 (47.1)
No	189 (52.9)
**Pit latrine without cement slab**	
Yes	38 (10.6)
No	319 (89.4)
**No toilet (canal, open defecation, bush, field)**	
Yes	5 (1.4)
No	352 (98.6)
**Toilet facilities: others**	
Yes	3 (0.8)
No	354 (99.2)
**Toilet facilities shared with other households**	
Yes	182 (51.0)
No	175 (49.0)
**Washing of hands during the day**	

For peer review only - http://bmjopen.bmj.com/site/about/guidelines.xhtml

Yes	349 (97.8)
No	8 (2.2)
**Washing of hands after using the toilet**	
Yes	349 (98.3)
No	6 (1.7)
**Washing hands before eating**	
Yes	319 (91.4)
No	30 (8.6)
**Washing hands after eating (n=319 who answered yes above)**	
Yes	268 (76.8)
No	81 (23.2)
**Washing hands before cooking**	
Yes	194 (55.6)
No	155 (44.4)
**Washing hands after cleaning tables**	
Yes	79 (22.6%)
No	270 (77.4%)
**Washing hands after cleaning baby stool/diapers**	
Yes	113 (32.4)
No	236 (67.6)
**Washing hands after cleaning house**	
Yes	120 (34.4)
No	229 (65.6)
**Washing hands any time**	
Yes	67 (19.2)
No	282 (80.8)
**Washing hands any other time**	
Yes	4 (1.1)
No	345 (98.9)

Analysis of respondents who received the first and second OCV doses (n=1, 691)

Out of the total sample, 1, 375 (81.3%) received the first dose. Of these, 994 (58.8%) were female, 185 (10.9%) were aged between 1 and 4 years, 637 (37.7%) between 5 and 7 years, 543 (32.1%) between 18 and 35 years, 285 (16.9%) were aged between 36 and 65; 41 (2.4%) were aged above 65 years. The differences in characteristics between the respondents who received and those who did not receive the OCV during the first round are shown table 3 below (**Table 3**).

Table 3: Respondents who received the first and second OCV doses

For peer review only - http://bmjopen.bmj.com/site/about/guidelines.xhtml

First OCV Dose			
**Variable**	**Yes (n=1, 375)**	**No (n=316)**	**Total (n=1, 691)**
	**n(%)**	**n (%)**	
**Sex**			
Male	556 (40.4)	141 (20.2)	697 (41.2)
Female	819 (59.6)	175 (17.6)	994 (58.8
**Age (in years)**			
1-4	158 (11.5)	27 (8.5)	185 (10.9)
5-14	554(40.3)	83(26.3)	637 (37.7)
15-35	409 (29.7)	134 (42.4)	543 (32.1)
36-65	226 (16.4)	59 (18.7)	285 (16.9)
Above 65	28 (2.1)	13 (4.1)	41 (2.4)
**Second dose**			
Variable	Yes (n=1, 220)	No (n=471)	Total (n=1, 691)
	n(%)	n (%)	
Sex			
Male	476 (39.0)	234 (49.6)	710 (42.0)
Female	744 (61.0)	237 (50.4)	981(58.0)
Age			
1-4	143 (11.7)	46 (9.8)	189 (11.1)
5-14	520 (42.6)	159 (33.8)	679 (40.2)
15-35	338 (27.7)	172 (36.5)	510 (30.2)
36-65	189 (15.5)	87 (18.4)	276 (16.3)
Above 65	30 (2.5)	7 (1.5)	37 (2.2)

Analysis of respondents who received the second OCV doses (n=1, 691)

Out of the total sample of 1,691 that was included in the analysis for the second round of the OCV campaign, 1, 220 (72.1%) received the second dose. Of these, 744 (61.0%) were female, 143 (11.7%) were aged between 1 and 4 years, 520 (42.6%) were between 5 and 14 years, 338 (27.7%) were between 15 and 35 years, 189 (15.5%) were aged between 36 and 65; 30 (2.5%) were aged above 65 years (**table 4**).

Table 4: Respondents who received the first and second OCV dose

Second OCV Dose			
**Variable**	Yes (n=1, 220)	No (n=471)	Total (n=1, 691)
	n(%)	n (%)	
Sex			
Male	476 (39.0)	234 (49.6)	710 (42.0)
Female	744 (61.0)	237 (50.4)	981(58.0)
Age			
1-4	143 (11.7)	46 (9.8)	189 (11.1)

For peer review only - http://bmjopen.bmj.com/site/about/guidelines.xhtml

5-14	520 (42.6)	159 (33.8)	679 (40.2)
15-35	338 (27.7)	172 (36.5)	510 (30.2)
36-65	189 (15.5)	87 (18.4)	276 (16.3)
Above 65	30 (2.5)	7 (1.5)	37 (2.2)

Administrative coverage

The overall administrative coverage for the OCV first dose was 110%. Kanyama sub-district reported the highest coverage at 141.9%, followed by Chipata at 124.1%, Chawama at 98.4%; Matero sub-district had the lowest at 82.4%. The overall coverage for the second dose was 89%. Kanya sub-district recorded the highest coverage at 158%, followed by Chawama at 98%, Chipata at 63%. Matero recorded the lowest coverage at 62.9%

OCV campaign coverage by dose based on the survey (n= 1691)

Table 5 below shows the OCV campaign coverage for each dose based on the survey results. The vaccine coverage for the first dose was 81.3%, (95%CI [79.24, 83.36]); the coverage for the second dose was 72.1% (95%CI [69.58, 74.62]); the two-dose coverage was 66% (95% CI [63.22, 68.78]). The drop-out rate was 18.8% (95%CI [14.51, 23.09]) (**Table 5**).

Table 5: OCV campaign coverage by dose

OCV dose	Number vaccinated	% (95% Confidence Interval)
First dose	1375	81.3 [79.24 – 83.36]
Second dose	1220	72.1 [69.58 –74.62]
Both doses	1116	66 [63.22 – 68.78]
Drop-out rate	318	18.8 [14.51 – 23.09]
At least one dose*	363	21.5 [17.27 – 25.73]

*Excludes those that received both doses

Discussion

The aim of this study was to determine the actual coverage (based on the population vaccinated) for the OCV campaign that was conducted in Lusaka district in 2018. Overall, our findings showed that the reported administrative coverage was much higher than the actual coverage. Coverage for the two-doses was 66.0%; coverage for the first and second dose was 81.3% and 72.1%, respectively. The drop- out rate was 18.8%.

For peer review only - http://bmjopen.bmj.com/site/about/guidelines.xhtml

For both the first and second campaigns, majority of the participants who received the vaccine were female in the age range 5 and 14 years (42.6%) and 15 35 years (27.7%). Only 15.5% and 2.5% of the participants aged between 36 and 65 and above 65 years, respectively, received the second dose.

These findings show that the administrative coverage reported during the first and second rounds was much higher than the actual one from the population survey. These findings are consistent with a survey conducted by Semá and colleagues [25] in Mozambique which reported a difference between the OCV administrative coverage and the one from the population survey. According to these authors, the administrative coverage provided by the Mozambican MoH were 108.4% and 107.9% after the first and second rounds, respectively. However, based on the population survey, the overall vaccination coverage with at least one dose was 69.5% (95%CI: 51.2–88.2); the full dose coverage (two-dose) was 51.2% (95%CI: 37.9–64.3). The discrepancy between the administrative and survey coverages could be due to data quality challenges and low population denominators. For example, in their study on monitoring vaccination coverage, Cutts and colleagues [26] showed that the population denominator and the reported number of vaccinations used in administrative estimates are often inaccurate. These authors concluded that, because of the inaccuracy associated with administrative data, surveys are often considered to be more accurate. Reliance on health facility data alone for calculating the OCV campaign coverage may mask low coverage and prevent subsequent measures to improve programming. Thus, there is need for periodic validation of administrative coverage estimates with population-based methods [27].

Our findings provide evidence on the feasibility of conducting an OCV campaign during a cholera outbreak according to the WHO guidelines which require conducting OCV campaign during an outbreak in addition to traditional interventions. The findings also corroborate previous studies which reported OCV campaign coverage using population survey data. For example, a survey conducted by Bwire and colleagues [28] on the use of surveys to evaluate an integrated oral cholera vaccine campaign in response to a cholera outbreak in Hoima district, Uganda, reported a (94% (95% CI 92% to 95%) coverage for at least one OCV dose and 78% (95% CI 76% to 81%) for two doses. Similarly, a Malawian survey by Sauvageot and colleagues [29] reported a high 2-dose coverage of 92.2% among participants who lived within 2km of lake Chilwa. In Iraq, Lam et al [30] reported an overall 2-dose OCV coverage of 87% in the targeted camps. Other studies have reported low coverage. A study conducted in Guinea by Luquero and others [31] reported a 2-dose coverage

For peer review only - http://bmjopen.bmj.com/site/about/guidelines.xhtml

of 75.5%. In a Mozambican survey, Semá and colleagues reported an overall 2-dose coverage of 51.2% and 69.5% for at least one dose.

Our findings show a vaccine drop-out rate of 18.8%. This finding is contrary to the WHO guidelines which recommend that, to be protected, an individual needs to receive two doses of Euvichol-Plus® [32]. The WHO guidelines also recommend that there must be a minimum of two weeks delay between two doses. Two doses of Euvichol-Plus® provide protection against cholera at least for three years, while one dose provides short term protection [33]. Similarly, Bekolo and colleagues [34] showed that patients who receive two doses of the OCV are 4.5 times less likely to develop severe disease than unvaccinated patients. Thus, individuals who receive only one dose do not get optimal protection from the vaccine. Future studies should focus on factors contributing to vaccine drop out after an individual has received the initial dose. Addressing these factors would increase coverage for both the first and second dose and maximize population benefits from the vaccine.

Finally, our findings show that majority of participants were females in the age range 5 to 14 and 15 to 35. The lowest coverage was among the older people aged above 65 years. These findings contradict those reported by Chitio and colleagues [35] which showed low vaccination coverage among Mozambican female respondents. The reason for this discrepancy is not clear; it could be due to sampling variation. Studies conducted on health service utilization have reported high utilization rates among females [36]. For example, Sialubanje and colleagues [37] reported a low COVID-19 vaccine coverage among males in Zambia. The main reason for the vaccine hesitancy and low coverage were alcohol, payer, traditional remedies, limited understanding and misconceptions, adverse effects and limited information.

These findings are consistent with those reported in a post OCV campaign survey by Semá and colleagues in Mozambique which reported the highest coverage for the two-doses among adult females and lowest among adult males. Our findings are also in line with those reported by Burnett and colleagues [38] in their study on the oral cholera vaccination coverage after the first global stockpile deployment in Haiti in 2014. These authors reported a higher percentage of children aged 5–14 years who received both recommended doses than did those ≥ 15 years. Public Health interventions planning OCV campaigns should take into consideration these sex and age differences in vaccine coverages in order to achieve optimal vaccine coverage

Limitations

For peer review only - http://bmjopen.bmj.com/site/about/guidelines.xhtml

Potential limitations for this study should be acknowledged. First, although a random satellite-based sampling technique was used to select households, inclusion of all eligible participants found in a household into the study may have introduced selection bias. Second, our findings are based on a survey conducted following an OCV campaign in four urban sub-districts of Lusaka City. The generalizability of these findings to other settings within the country may be limited.

Despite these limitations, we believe this study has provided evidence on the actual coverage of the OCV campaign based on the survey and clearly shown that the reported administrative coverage was much higher than the actual one from the survey. These findings provide evidence on the importance of validating administrative vaccination campaign coverage with survey data based on the actual vaccinated population. Moreover, our findings confirm the feasibility of conducting an OCV campaign and achieving high coverage during a cholera outbreak in an urban setting like Lusaka city [39]. They corroborate previous campaigns and surveys conducted in other countries as well as the WHO guideline which recommend complementing traditional cholera control and preventions strategies (provision of safe drinking water, adequate sanitation, disease surveillance, health promotion and case management) with OCV campaign during an outbreak.

Conclusion

These findings show that the 2018 OCV campaign coverage reported using administrative data was much higher than the actual one based on the population that was vaccinated. Follow up surveys are needed to validate administrative coverage estimates. Reliance on health facility data alone may mask low coverage and prevent measures to improve programming. The findings also confirm the feasibility of conducting an OCV campaign during a cholera outbreak in an urban setting. Future studies should focus on determining the long term effectiveness of single and two-doses in the population as well as their cost-effectiveness.

Author contribution

All authors contributed substantially to the development of this manuscript. VM, CS, NK, BM, OC ANM, WN, MK, LM, RH, HM and NS contributed to the conception of the study. NK, BM, OC, NS, HM and LC supervised the data collection process. WN, CS and RH conducted the data analysis. CS, HM, OC and BM drafted the

For peer review only - http://bmjopen.bmj.com/site/about/guidelines.xhtml

manuscript; BM and VM edited it; CS revised the manuscript. All authors read, commented on and approved the final manuscript. CS is the guarantor for the manuscript.

Funding Statement

Ministry of Health and World Health Organization (award/grant number: N/A)

Competing Interests

All authors declare that they have no conflicting interests in this work

Data Sharing

Data are available upon reasonable request from the corresponding author and with permission of the ERES ethics review board.

Costs and payments

There was no cash payment provided to participants for any portion of the study. Participants volunteered their time taken to participate in the study. As a small token of appreciation for their time and opportunity costs, participants received a bottle of water or soft drink, in line with the ERES guideline.

Acknowledgements

We thank Lusaka District Health Management Team who provided field research assistants and study participants. The GTFCC supported the country with OCV stocks and operations funds.

Ethics approval

Ethical approval for the study was obtained from the Excellence in Research Ethics and Science (ERES, 2019-09-001) Converge Committee and authority to conduct the study was obtained from the National Health Research Authority. Written informed consent was obtained from all participants in the study.

Figures

For peer review only - http://bmjopen.bmj.com/site/about/guidelines.xhtml

For peer review only

Figure 1: Recruitment algorithm

References

1. Mukhopadhyay A.K., Takeda Y., Balakrish Nair. Cholera Outbreaks in the El Tor Biotype Era and the Impact of the New El Tor Variants. In: Nair, G., Takeda, Y. (eds) Cholera Outbreaks. Current Topics in Microbiology and Immunology, 2014; vol 379. Springer, Berlin, Heidelberg. https://doi.org/10.1007/82_2014_363

2. Son MS, Megli CJ, Kovacikova G, Qadri F, Taylor RK. Characterization of Vibrio cholerae O1 El Tor biotype variant clinical isolates from Bangladesh and Haiti, including a molecular genetic analysis of virulence genes. J Clin Microbiol; 2011;49(11):3739-49. doi: 10.1128/JCM.01286-11.

3. Deen J, Mengel MA, Clemens JD. Epidemiology of cholera. Vaccine, 2020;38 Suppl 1:A31-A40. doi: 10.1016/j.vaccine.2019.07.078

4. Rosewell A, Addy B, Komnapi L, Makanda F, Ropa B, Posanai E et al. Cholera risk factors, Papua New Guinea, 2010. BMC Infect Dis, 2012; 12:287. doi: 10.1186/1471-2334-12-287

5. WHO, 2022. Cholera. Available at: https://www.who.int/news-room/fact-sheets/detail/cholera, accessed on 10th July, 2022

6. CDC, 2022. About Global Cholera. Available at: About Global Cholera (cdc.gov), accessed on 1st December, 2022

For peer review only - http://bmjopen.bmj.com/site/about/guidelines.xhtml

7. CDC, 2022. Data and Statistics on Global Cholera. Available at: Data and Statistics on Global Cholera (cdc.gov), accessed on 10th August, 2023

8. WHO, 2011. WHO Consultation on oral cholera vaccine (OCV) stockpile strategic framework: potential objectives and possible policy options. Available on the Internet at: www.who.int/vaccines-documents, accessed on 10th August, 2023

9. Lopez AL, Gonzales ML, Aldaba JG, Nair GB. Killed oral cholera vaccines: history, development and implementation challenges. Ther Adv Vaccines. 2014 Sep;2(5):123-36. doi: 10.1177/2051013614537819. PMID: 25177492; PMCID: PMC4144262

10. WHA. Stop Cholera. Available at: The 64th World Health Assembly Resolution for Renewed Efforts to Prevent and Control Cholera (May 2011) | DOVE: Stop Cholera, accessed on 8th August, 2023

11. WHO. Cholera vaccine. Available at: Immunization, Vaccines and Biologicals (who.int), accessed on 8th August, 2023

12. Bi Q, Ferreras E, Pezzoli L, Legros D, Ivers LC, Date K, et al. Oral Cholera Vaccine Working Group of The Global Task Force on Cholera Control. Protection against cholera from killed whole-cell oral cholera vaccines: a systematic review and meta-analysis. Lancet Infect Dis. 2017; 10:1080-1088. doi: 10.1016/S1473-3099(17)30359-6.

13. Franke MF, Ternier R, Jerome JG, Matias WR, Harris JB, Ivers LC. Long-term effectiveness of one and two doses of a killed, bivalent, whole-cell oral cholera vaccine in Haiti: an extended case-control study. Lancet Glob Health. 2018 Sep;6(9):e1028-e1035. doi: 10.1016/S2214-109X(18)30284-5.

For peer review only

For peer review only - http://bmjopen.bmj.com/site/about/guidelines.xhtml

14. Franke MF, Jerome JG, Matias WR et al. Comparison of two control groups for estimation of oral cholera vaccine effectiveness using a case-control study design. *Vaccine.* 2017; 35: 5819-582

15. Wierzba TF, Kar SK, Mogasale VV et al. Effectiveness of an oral cholera vaccine campaign to prevent clinically-significant cholera in Odisha State, India. *Vaccine.* 2015; 33: 2463-2469

16. Kanungo S, Sen B, Ramamurthy T, Sur D, Manna B, Pazhani GP et al. Safety and immunogenicity of a live oral recombinant cholera vaccine VA1.4: a randomized, placebo controlled trial in healthy adults in a cholera endemic area in Kolkata, India. PLoS One, 2014; 1;9(7):e99381. doi: 10.1371/journal.pone.0099381.

17. Baik YO, Choi SK, Olveda RM, Espos RA, Ligsay AD, Montellano MB et al. A randomized, non-inferiority trial comparing two bivalent killed, whole cell, oral cholera vaccines (Euvichol vs Shanchol) in the Philippines. Vaccine, 2015; 17;33(46):6360-5. doi: 10.1016/j.vaccine.2015.08.075

18. Poncin M, Zulu G, Voute C, Ferreras E, Muleya MC, Malama K et al. Implementation research: reactive mass vaccination with single-dose oral cholera vaccine, Zambia. Bull World Health Organ, 2018;96:86–93 | doi: http://dx.doi.org/10.2471/BLT.16.18924

19. WHO, 2017. African Region. Available at: Disease outbreak - Cholera - Zambia - 11 December 2017 | WHO | Regional Office for Africa, accessed on 8th August, 2023

20. WHO 2021. Mass Cholera Vaccination Campaign Launched. Available at: Mass cholera vaccination campaign launched (who.int), accessed on 8th August, 2023

21. Sialubanje C, Kapina M, Chewe O*, et al.* Effectiveness of two doses of Euvichol-plus oral cholera vaccine in response to the 2017/2018 outbreak: a matched case–control study in Lusaka, Zambia. *BMJ Open* 2022;12:e066945. doi: 10.1136/bmjopen-2022-066945

For peer review only - http://bmjopen.bmj.com/site/about/guidelines.xhtml

22. Massar K, Sialubanje C, Maltagliati I and Ruiter ACR. Exploring the Perceived Effectiveness of Applied Theater as a Maternal Health Promotion Tool in Rural Zambia. Qualitative Health Research, 2018; 28(12) 1933–1943

23. Sialubanje C, Sitali DC, Mukumbuta N*, et al.* Perspectives on factors influencing transmission of COVID-19 in Zambia: a qualitative study of health workers and community members. *BMJ Open* 2022;12:e057589. doi: 10.1136/bmjopen-2021-057589

24. Central Statistics Office, Ministry of Health and ICF International. Zambia Demographic and Health Survey 2013-14. Rockville, Maryland, USA; 2014.

25. Semá Baltazar SC, Rafael F, Langa JPM, Chicumbe S, Cavailler P, Gessner BD, et al. Oral cholera vaccine coverage during a preventive door-to-door mass vaccination campaign in Nampula, Mozambique. PLoS One. 2018 Oct 3;13(10):e0198592. doi: 10.1371/journal.pone.0198592.

26. Cutts FT, Claquin P, Danovaro_Holliday MC and Rhoda DA. Monitoring vaccination coverage: defining the role of surveys. Vaccine, 2016; 34 (35): 4103-4109

27. Janmohamed A, Doledec D. Comparison of administrative and survey data for estimating vitamin A supplementation and deworming coverage of children under five years of age in Sub-Saharan Africa. Trop Med Int Health. 2017;22(7):822-829. doi: 10.1111/tmi.12883. Epub 2017 May 26. PMID: 28449319

For peer review only - http://bmjopen.bmj.com/site/about/guidelines.xhtml

28. Bwire G, Roskosky M, Ballard A, Brooks WA, Okello A, Rafael F et al. Use of surveys to evaluate an integrated oral cholera vaccine campaign in response to a cholera outbreak in Hoima district, Uganda. BMJ Open. 2020 Dec 10;10(12):e038464. doi: 10.1136/bmjopen-2020-038464.

29. Sauvageot D, Saussier C, Gobeze A, Chipeta S, Mhango I, Kawalazira G, et al. Oral cholera vaccine coverage in hard-to-reach fishermen communities after two mass Campaigns, Malawi, 2016. Vaccine,2017; 12;35(38):5194-5200. doi: 10.1016/j.vaccine.2017.07.104. Epub 2017 Aug 10. PMID: 28803712.

30. Lam E, Al-Tamimi W, Russell SP, Butt MO, Blanton C, Musani AS, Date K. Oral Cholera Vaccine Coverage during an Outbreak and Humanitarian Crisis, Iraq, 2015. Emerg Infect Dis. 2017;23(1):38-45. doi: 10.3201/eid2301.160881.

31. Luquero FJ, Grout L, Ciglenecki I, Sakoba K, Traore B, Heile M et al. First outbreak response using an oral cholera vaccine in Africa: vaccine coverage, acceptability and surveillance of adverse events, Guinea, 2012. PLoS Negl Trop Dis. 2013;7(10):e2465. doi: 10.1371/journal.pntd.0002465.

32. WHO, 2022. Preventing and controlling cholera in endemic countries. Available at: Preventing and controlling cholera in endemic countries (who.int), accessed on 8th August, 2023

33. Desai SN, Pezzoli L, Martin S, Costa A, Rodriguez C, Legros D, Perea W. A second affordable oral cholera vaccine: implications for the global vaccine stockpile. Lancet Glob Health. 2016 Apr;4(4):e223-4. doi: 10.1016/S2214-109X(16)00037-1. PMID: 27013303

34. Bekolo CE, JAF van Loenhout, Rodriguez-Llanes, JM, Rumunu J, Ramadanc OP & Guha-Sapir D. A retrospective analysis of oral cholera vaccine use, disease severity and deaths during an

For peer review only - http://bmjopen.bmj.com/site/about/guidelines.xhtml

outbreak in South Sudan. Bull World Health Organ 2016;94:667–674 | doi: http://dx.doi.org/10.2471/BLT.15.166892

35. Elias Chitio JJ, Baltazar CS, Langa JP, et al. Pre-emptive oral cholera vaccine (OCV) mass vaccination campaign in Cuamba District, Niassa Province, Mozambique: feasibility, vaccination coverage and delivery costs using CholTool BMJ Open 2022;12:e053585. doi: 10.1136/bmjopen-2021-053585

36. Pugliese-Garcia M, Heyerdahl L, Mwamba C, Nkwemu S, Chilengi R, Demolis R et al. Factors influencing vaccine acceptance and hesitancy in three informal settlements in Lusaka, Zambia. Vacine, 2018; 36 (37); 5617-5624.

37. Sialubanje C, Mukumbuta N, Ng'andu M, et al. Perspectives on the COVID-19 vaccine uptake: a qualitative study of community members and health workers in Zambia. BMJ Open 2022;12:e058028. doi: 10.1136/bmjopen-2021-058028

38. Burnett EM, Francois J, Sreenivasan N, Wannemuehler K, Faye PC, Tohme RA et al. Oral cholera vaccination coverage after the first global stockpile deployment in Haiti, 2014. Vaccine. 2019 Oct 8;37(43):6348-6355. doi: 10.1016/j.vaccine.2019.09.018. Epub 2019 Sep 11. PMID: 31521413.

39. Vogel L. Mass cholera vaccination finally possible. CMAJ.2015; 187(7):E207-E208. doi: 10.1503/cmaj.109-5021

For peer review only - http://bmjopen.bmj.com/site/about/guidelines.xhtml

Figure 1: Recruitment algorithm

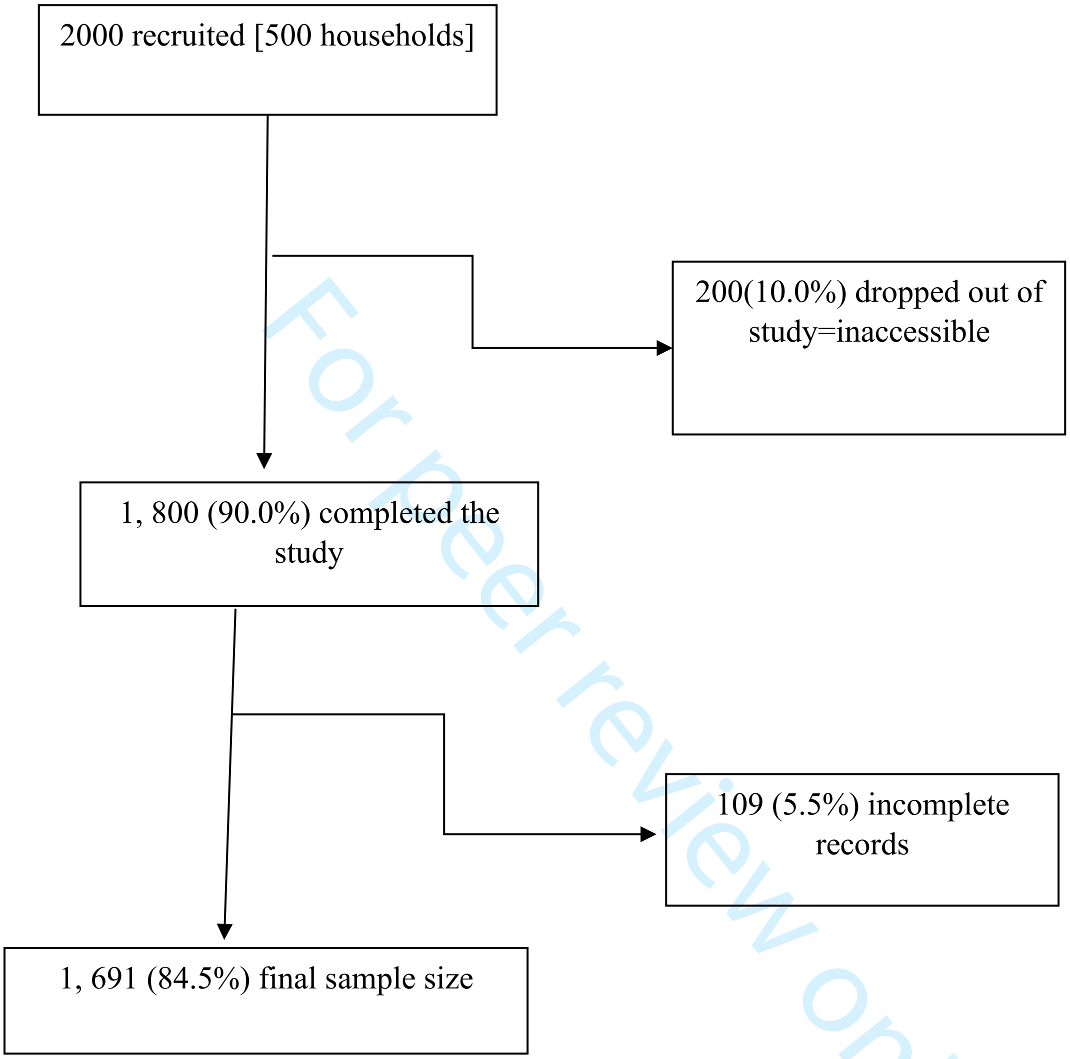

STROBE Statement—Checklist of items that should be included in reports of cohort studies

Item	Recommendation	Page
Title and abstract	Indicate the study's design with a commonly used term in the title or the abstract	1
	Provide in the abstract an informative and balanced summary of what was done and what was found	2
Introduction		
Background/rationale	Explain the scientific background and rationale for the investigation being reported	4
Objectives	State specific objectives, including any pre-specified hypotheses	5
Methods		
Study design	Present key elements of study design early in the paper	5
Setting	Describe the setting, locations, and relevant dates, including periods of recruitment, exposure, follow-up, and data collection	5
Participants	Give the eligibility criteria, and the sources and methods of selection of participants. Describe methods of follow-up	5
Study size	Explain how the study size was arrived at	6
Data sources/ measurement	For each variable of interest, give sources of data and details of methods of assessment (measurement). Describe comparability of assessment methods if there is more than one group	6
Bias	Describe any efforts to address potential sources of bias	7
Statistical methods	Describe all statistical methods, including those used to control for confounding	8
	If applicable, explain how loss to follow-up was addressed	
Patient and public involvement		8
Results		8
Discussion		14
Limitations		17
Conclusion		17
Funding		18
Ethical considerations		18
References		19

For peer review only
